# The short isoform of extended synaptotagmin-2 controls Ca^2+^ dynamics in T cells via interaction with STIM1

**DOI:** 10.1038/s41598-020-71489-7

**Published:** 2020-09-02

**Authors:** Jin Seok Woo, Zuoming Sun, Sonal Srikanth, Yousang Gwack

**Affiliations:** 1 Department of Physiology, David Geffen School of Medicine at UCLA, 53-266 CHS, 10833 Le Conte Avenue, Los Angeles, CA 90095 USA; 2 Department of Molecular Imaging & Therapy, Beckman Research Institute of City of Hope, Duarte, CA 91010 USA

**Keywords:** Molecular biology, Physiology

## Abstract

Ca^2+^ release-activated Ca^2+^ (CRAC) channels elevate cytoplasmic Ca^2+^ concentration, which is essential for T cell activation, differentiation and effector functions. T cell receptor stimulation induces depletion of the endoplasmic reticulum (ER) Ca^2+^ stores, which is sensed by stromal interaction molecule 1 (STIM1). STIM1 translocates to the ER-plasma membrane (PM) junctions to interact with ORAI1, the pore subunit of the CRAC channels. Here, we show that two members of the extended synaptotagmin (E-Syt) family, E-Syt1, and the short isoform of E-Syt2 (E-Syt2S), contribute to activation of CRAC channels in T cells. Knockdown or deletion of both *ESYT1* and *ESYT2* reduced store-operated Ca^2+^ entry (SOCE) and ORAI1-STIM1 clustering in Jurkat T cells. Further, depletion of E-Syts in primary T cells decreased Ca^2+^ entry and cytokine production. While the ER-PM junctions were reduced in both HeLa and Jurkat T cells deleted for *ESYT1* and *ESYT2*, SOCE was impaired only in Jurkat T cells, suggesting that the membrane-tethering function of E-Syts is distinct from their role in SOCE. Mechanistically, E-Syt2S, the predominant isoform of E-Syt2 in T cells, recruited STIM1 to the junctions via a direct interaction. This study demonstrates a membrane-tethering-independent role of E-Syts in activation of CRAC channels in T cells.

## Introduction

Ca^2+^ release-activated Ca^2+^ (CRAC) channels mediate a sustained increase in cytoplasmic Ca^2+^ concentration that is essential for T cell activation. T cell receptor stimulation induces the depletion of the endoplasmic reticulum (ER) Ca^2+^ stores that activates CRAC channels via a process called store-operated Ca^2+^ entry (SOCE). STIM1 and ORAI1 are two essential components of CRAC channels. STIM1 is an ER-resident regulatory subunit that senses depletion of the ER Ca^2+^ stores, and the plasma membrane (PM)-resident ORAI1 is the pore subunit of CRAC channels. Upon store depletion, STIM1 multimerizes and translocates from the ER to the ER-PM junctions (a space of 10–15 nm) via passive diffusion and interacts with ORAI1 to induce its opening^1–5^. The C-terminal poly(K) residues of STIM1 interact with phosphatidylinositol 4,5-bisphosphate (PIP_2_) in the PM for its recruitment to the ER-PM junctions. Further, protein interactions also play a crucial role for STIM1 recruitment into the junctions. We recently identified junctional proteins, including junctate and junctophilins, as important components of the ER-PM junctions in T cells^6,7^. Junctate-junctophilin complex is localized at the ER-PM junctions, and after sensing ER Ca^2+^ depletion via its ER-luminal Ca^2+^-binding motif, junctate recruits STIM1 into the junctions. Other molecules such as TMEM110 (alternatively, STIM-activating enhancer) and SARAF (SOCE-associated regulatory factor) have also been identified as components of the ER-PM junctions in T cells^8–10^. All these molecules interact with STIM1 to modulate its function, including translocation into the junctions (e.g., junctate and junctophilin-4) and induction of conformational changes (e.g., TMEM110 and SARAF). Other than this, our current understanding of the structural and regulatory components of the ER-PM junctions involved in Ca^2+^ signaling in T cells is limited.

The ER-PM junctions are ubiquitous structures essential for intermembrane communications, including lipid transfer and Ca^2+^ dynamics^11^. Yeast proteins, tricalbins (Tcb1p, Tcb2p, and Tcb3p) are selectively concentrated in the cortical ER and play an essential role in ER-PM tethering. Their mammalian homologs are E-Syt1, E-Syt2, and E-Syt3. E-Syts contain an ER membrane integration segment, a cytosolic synaptotagmin-like mitochondrial lipid-binding protein (SMP) domain followed by multiple PIP_2_ and Ca^2+^-binding C2 domains. SMP domains of E-Syts have a specialized function in lipid exchange^12,13^. E-Syt2 and E-Syt3 are ER-resident proteins that constitutively interact with the PM while E-Syt1 requires elevation of cytosolic Ca^2+^ (low micromolar range) for interaction with the PM^14,15^. E-Syt1 and STIM1 share at least partially the same contact sites, and it was shown that E-Syt1 is important for PIP_2_ enrichment and replenishment at the ER-PM junctions that are essential for STIM1 recruitment^16–18^. However, E-Syt-dependent contacts were not required for SOCE, at least in HeLa cells, as reducing their expression did not affect this process^15^.

Here we show that E-Syt1 and a short isoform of E-Syt2, E-Syt2S, have a compensatory role in CRAC channel activation in T cells. In addition to their conserved membrane-tethering function, E-Syt1 and E-Syt2S affect Ca^2+^ signaling in T cells by regulating the recruitment of STIM1 to the ER-PM junctions. Among the E-Syt2 isoforms, only E-Syt2S supported STIM1 translocation to the junctions, and is uniquely abundant in T cells. Accordingly, loss of E-Syt expression impairs SOCE and cytokine expression in those cells. Our results show that the ER-PM junctions in T cells consist of unique members (i.e., E-Syt2S) that play specialized roles in regulation of Ca^2+^ signaling.

## Results

### Identification of E-Syts as important regulators of CRAC channels

**Figure 1:**
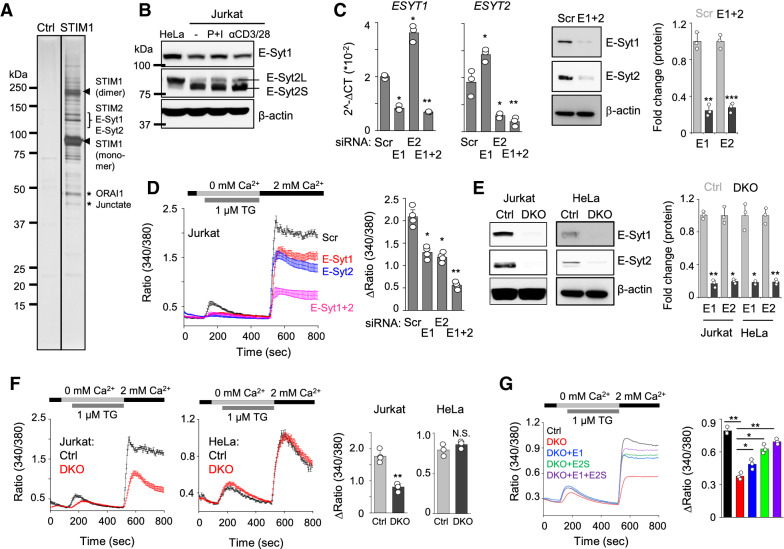
Identification of E-Syts as regulators of CRAC channels. (**A**) Affinity purification of STIM1 protein complex. The glycerol gradient fractions enriched in FLAG-STIM1 were pooled and immunoprecipitated with anti-FLAG resin. After elution with the FLAG peptide, fractions were separated on SDS–PAGE and visualized by silver staining. Asterisks and bracket indicate bands from which STIM2, E-Syt1, E-Syt2, junctate, and ORAI1 were identified by mass spectrometry. Arrowheads indicate potential monomeric and dimeric forms of STIM1. (**B**) Jurkat T cells were stimulated with PMA (80 nM) and ionomycin (1 µM, P + I) or anti-CD3/28 (each 10 µg) antibodies for 6 h. Lysates from unstimulated HeLa cells and unstimulated/stimulated Jurkat T cells were subjected to immunoblot analysis to detect E-Syt1 and E-Syt2. β-actin was used as a loading control. (**C**) Bar graphs show mRNA levels of *ESYT1* and *ESYT2* in Jurkat T cells expressing scrambled siRNA (Scr) or those targeting *ESYT1* (E1), *ESYT2* (E2), or both (E1 + 2) (left). Representative immunoblots show expression of E-Syt1 and E-Syt2 proteins (middle) in indicated cells. Bar graph shows normalized protein levels of E-Syt1 and E-Syt2 (right). **p < 0.005, ***p < 0.0005. (**D**) SOCE measurement in Jurkat T cells expressing scrambled shRNA or shRNA targeting transcripts of *ESTY1*, *ESYT2*, or both (left). Intracellular stores were passively depleted with thapsigargin (1 μM, TG) in Ca^2+^-free Ringer’s solution, and SOCE was measured by perfusion with 2 mM Ca^2+^-containing Ringer’s solution. (**E**) Representative immunoblots showing expression of E-Syt1 and E-Syt2 in control (Ctrl) or *ESYT1/ESYT2* double knockout (DKO) HeLa or Jurkat T cells. Bar graph shows normalized protein levels of E-Syt1 and E-Syt2 (right). *p < 0.05, **p < 0.005. (**F**) SOCE measurements from control and DKO Jurkat T and HeLa cells as described above (**D**). (**G**) Measurement of SOCE in control, DKO, and DKO Jurkat T cells expressing E-Syt1 (E1), E-Syt2S (E2S) or both (E1 + E2S) as indicated above (**D**). In (**D**,**F**,**G**), traces show averaged SOCE responses from 30 to 50 cells, and bar graphs show averaged SOCE response (peak – basal) ± S.E.M. from three independent experiments. *p < 0.05, **p < 0.005.

To identify components of the ER-PM junctions where ORAI1 and STIM1 interact, we carried out large-scale protein affinity purification using cells stably expressing low levels of FLAG-tagged STIM1 (Fig. 1A). This purification consisted of multiple steps of large-scale cell culture, crosslinking to capture both the stable and transient protein complexes, enrichment of the complexes with glycerol gradient fractionation, and finally affinity purification using anti-FLAG antibody-conjugated resin^19^. The eluted protein complexes were run on an SDS-PAGE gel, and protein bands were cut and analyzed using nano-liquid chromatography with a tandem mass spectrometer (nLC-MS/MS). E-Syt1 and E-Syt2 were identified from the results of mass spectrometry in addition to the known interacting partners of STIM1, including ORAI1, the other STIM family member, STIM2, and junctate^6^.

Depletion of E-Syt1, E-Syt2, and E-Syt3 did not influence SOCE in HeLa cells^15^. However, the role of E-Syts in regulation of SOCE in T cells, where CRAC channels play a predominant role, has not been investigated. We first checked the expression of E-Syts in Jurkat T cells in comparison with HeLa cells (Fig. 1B). Both E-Syt1 and E-Syt2 were abundantly expressed in HeLa and Jurkat T cells, and their expression in Jurkat T cells was not influenced upon stimulation. E-Syt3 was not considered here because it was not identified in our affinity protein purification, and its transcripts were not detectable in Jurkat T cells. While E-Syt1 protein levels were slightly lower in Jurkat T cells, those of E-Syt2 were similar between the two cell types. Interestingly, the predominant form of E-Syt2 protein expressed in Jurkat T cells was of a smaller size than that in HeLa cells. Previously two isoforms of E-SYT2 differing by 48 amino acids have been described^15^. The larger protein comprising of 893 amino acids (NP_065779.1) is hereafter referred to as E-Syt2L, while the smaller protein of 845 amino acids (NP_065779.2) will be indicated as E-Syt2S^15^. To confirm that E-Syt2S was indeed the predominant isoform in Jurkat T cells, we compared the molecular weights of endogenous E-Syt2 proteins in HeLa and Jurkat T cells with exogenously expressed E-Syt2L and E-Syt2S proteins. Our immunoblot analysis showed that the molecular weight of exogenously expressed E-Syt2S matched that of the isoform abundant in Jurkat T cells, while that of E-Syt2L matched the isoform expressed abundantly in HeLa cells (Fig. S1A). Another long transcriptional isoform, *ESYT2a* (ABJ97706.1) encoding a protein of 921 amino acids, was described in a previous report^20^, but we could not detect its transcript or protein in HeLa and Jurkat T cells (Figs. 1B, S1B).

To determine the role of E-Syts in Jurkat T cells, we generated Jurkat T cells stably expressing shRNAs to deplete the expression of *ESYT1*, *ESYT2*, and both, and confirmed their depletion at transcript and protein levels (Fig. 1C). Interestingly, depletion of *ESYT1* increased transcript levels of *ESYT2* and vice versa, suggesting their functional redundancy in T cells. To check their role in CRAC channel regulation, we measured SOCE by depletion of the ER Ca^2+^ stores with thapsigargin, a sarco/endoplasmic reticulum Ca^2+^ ATPase (SERCA) blocker. While depletion of either E-Syt1 or E-Syt2 showed a modest, but statistically significant decrease in SOCE in Jurkat T cells, that of both the proteins showed a striking reduction in SOCE (Fig. 1D). Previous report showed that E-Syts are dispensable for SOCE in HeLa cells^15^. To confirm the distinct role of E-Syts in these two cell-lines, we generated HeLa and Jurkat T cells deleted for both *ESYT1* and *ESYT2* (double knockout cells, DKO) using the CRISPR/Cas9 system (Fig. 1E). In consistence with our knockdown analysis, we observed a significant decrease in SOCE in DKO Jurkat T cells while DKO HeLa cells did not show a reduction in SOCE (Fig. 1F). We observed reduced SOCE in DKO Jurkat T cells upon TCR stimulation as well (Fig. S1C). The specific and compensatory role of E-Syt1 and E-Syt2 in SOCE in Jurkat T cells was validated by the rescue of reduced SOCE upon exogenous expression of E-Syt1, E-Syt2S, and both. While expression of each of the E-Syts significantly rescued the SOCE defect in DKO cells, co-expression of both E-Syt1 and E-Syt2S showed maximal rescue of SOCE (Fig. 1G).

### Role of E-Syts in Ca^2+^ entry and cytokine production in T cells

**Figure 2:**
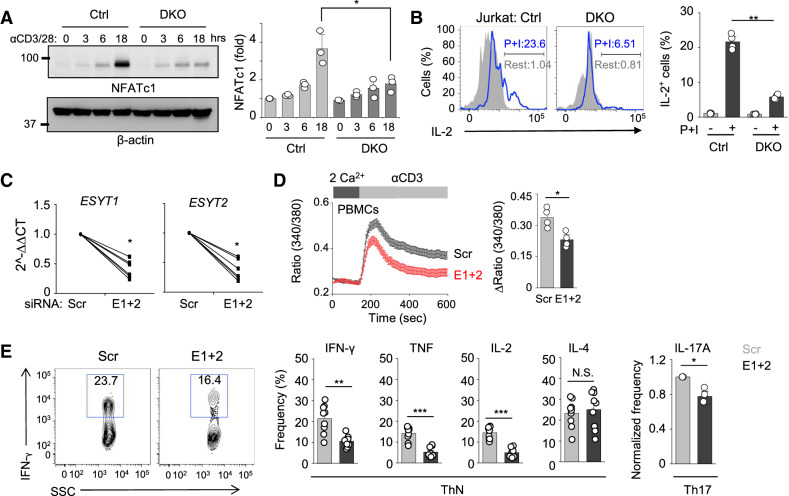
Deficiency of E-Syts impairs the NFAT pathway and cytokine production in T cells. (**A**) Representative immunoblot showing levels of NFATc1 from lysates of control and DKO Jurkat T cells stimulated with plate-coated anti-CD3 antibody (10 μg/ml) together with soluble anti-CD28 (10 μg/ml) for indicated times. β-actin was used as a loading control. Densitometry graph below shows normalized fold induction of NFATc1 from three independent experiments. **p < 0.005. (**B**) Representative flow plots showing IL-2 expression in control and DKO Jurkat T cells under resting conditions or after stimulation with PMA (80 nM) and ionomycin (1 μM; P + I) for 6 h. Bar graph below shows averaged percentage (± S.E.M.) of IL-2^+^ cells from four independent experiments. **p < 0.005. (**C**) Transcript levels of *ESYT1* and *ESYT2* in human PBMCs expressing control shRNA (Scr) and shRNAs targeting transcripts of *ESYT1* and *ESYT2* (E1 + 2). Each symbol represents an independent donor. *p < 0.05. (**D**) Measurement of SOCE in human PBMCs depleted of E-Syt1 and E-Syt2 after TCR cross-linking in the presence of external solution containing 2 mM Ca^2+^. Traces show averaged SOCE responses from 30 to 50 cells, and the bar graph shows averaged response ± S.E.M. from three independent experiments. *p < 0.05. (**E**) Representative flow plots and bar graphs showing expression of IFN-γ, TNF, IL-2, IL-4, and IL-17A in Scr and DKD human PBMCs differentiated under non-polarizing or Th17-polarizing conditions (see “Materials and methods" section) for 5 days and re-stimulated with PMA (40 nM) and ionomycin (1 µM) for 6 h. Each symbol represents an independent donor. *p < 0.05, **p < 0.005, ***p < 0.001, *N.S.* not significant.

To examine the functional outcome of reduced SOCE by deletion of *ESYT1* and *ESYT2* in T cells, we checked activation of the nuclear factor of activated T cells (NFAT) pathway. Previously, we showed that Ca^2+^-mediated activation of NFAT further induces expression of NFATc1, via a positive feedback mechanism^21^. We observed much reduced induction of NFATc1 in *ESYT* DKO cells after TCR stimulation as compared to control cells (Fig. 2A). To investigate physiological outcomes of reduced SOCE in *ESYT* DKO cells, we examined Ca^2+^-dependent cytokine production. Accordingly, we observed reduced IL-2 expression in DKO cells (Fig. 2B). Considering the broad role of E-Syts in cellular functions, including membrane tethering and glycerolipid transfer, we examined if deficiency of E-Syts influences TCR proximal signaling or other downstream signaling pathways, including phosphorylation of the signaling adaptor, ZAP70 as well as mitogen-activated protein kinases (MAPKs)—extracellular signal-regulated kinase (ERK), p38, and c-Jun N-terminal kinase (JNK). Our results showed that phosphorylation of all these TCR signaling molecules remained unaffected by deficiency of E-Syts (Fig. S2A). In addition, we also checked the PIP_2_ levels in control and DKO cells using a biomarker containing the pleckstrin homology (PH) domain of PLCδ that binds to PIP_2_ with high affinity (PLCδ-PH-eGFP). We did not observe any significant change in the distribution of PLCδ-PH-eGFP signals in DKO Jurkat T cells under resting conditions, suggesting that E-Syts are not necessary for homeostatic regulation of PIP_2_ at the plasma membrane (Fig. S2B). These results suggest that although E-Syts are known to have multiple functions, their deficiency in T cells primarily influences the Ca^2+^-NFAT pathway.

Next we checked if the role of E-Syts in Ca^2+^-NFAT signaling in Jurkat T cells was also conserved in primary human CD4^+^ T cells. Similar to Jurkat T cells, knockdown of E-Syt1 and E-Syt2 (average efficiency > 50%) significantly decreased SOCE in human effector CD4^+^ cells that were cultured under non-polarizing conditions (Fig. 2C,D). Consistently, expression of pro-inflammatory cytokines including IFN-γ, tumor necrosis factor (TNF), and IL-2 was reduced while that of IL-4 remained unchanged (Fig. 2E). Also, when cultured under Th17-polarizing conditions, human T cells depleted of both E-Syt1 and E-Syt2 showed reduced expression of IL-17A.

### Role of E-Syts in STIM1 translocation to the ER-PM junctions

**Figure 3:**
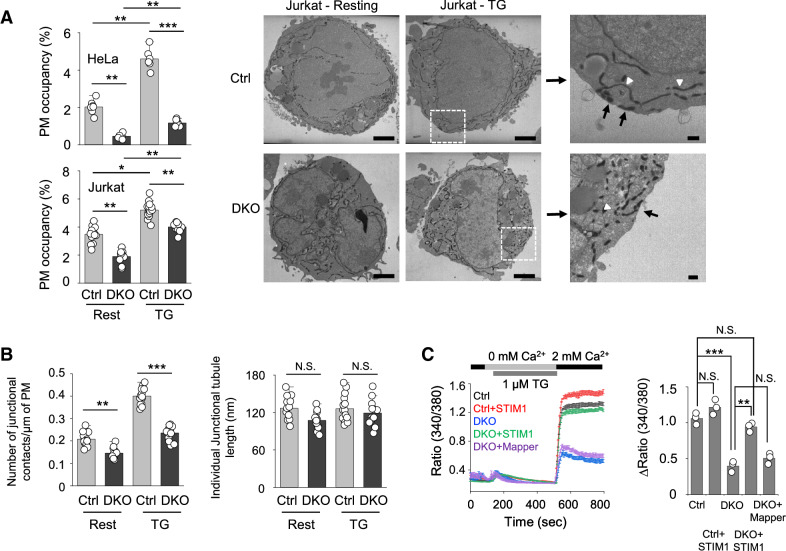
Function of E-Syts in endoplasmic reticulum membrane and plasma membrane tethering in T cells. (**A**) Bar graphs showing the PM occupancy of the junctional HRP-tubules in control and DKO HeLa (Ctrl rest: n = 7, Ctrl TG: n = 6, DKO rest: n = 6, DKO TG: n = 7) and Jurkat T (Ctrl rest: n = 13, Ctrl TG: n = 12, DKO rest: n = 15, DKO TG: n = 13) cells. Right panels show representative electron microscopy images of Jurkat T cells (resting and store depleted) expressing HRP-ER and processed for HRP cytochemistry showing ER tubules at the ER–PM junctions (black arrows) and in the cytoplasm (white arrowheads) (Scale bars, 2 μm; Inset, 0.2 μm). *p < 0.05, **p < 0.005, ***p < 0.001. (**B**) Bar graphs show number of junctional contacts/μm of the PM and length of the Individual Junctional-HRP tubules per section (normalized to the PM circumference) in control (rest: n = 13, TG: n = 12), and DKO (rest: n = 15, TG: n = 13) Jurkat T cells. **p < 0.005, ***p < 0.001. *N.S.* not significant. (**C**) SOCE measurement in control, DKO, and DKO Jurkat cells expressing STIM1 or MAPPER. Intracellular stores were passively depleted with thapsigargin (1 μM, TG) in Ca^2+^-free Ringer’s solution, and SOCE was measured by perfusion with 2 mM Ca^2+^-containing Ringer’s solution. Traces show averaged SOCE responses from 30 to 50 cells, and bar graph shows averaged SOCE response (peak – basal) ± S.E.M. from three independent experiments. *p < 0.05.

One plausible hypothesis for the selective role of E-Syts in SOCE in Jurkat T cells is that Jurkat T cells may be more dependent on E-Syt proteins to establish their ER-PM junctions as compared to HeLa cells. To check this possibility, we examined the PM occupancy and number of the ER-PM junctions in control and *ESYT* DKO Jurkat T cells in comparison with those in HeLa cells (Fig. 3A). Previous reports demonstrated a significant role of E-Syts in ER-PM tethering in HeLa cells, such that depletion of E-Syts decreased the ER-PM junctions by ~ 70%^15^. Consistently, we observed a more than 70% decrease in the PM occupancy of the junctions in *ESYT* DKO HeLa cells when compared to control cells. Also, in consistence with the previous reports^5,22^, we found that the PM occupancy of the ER-PM junctions was increased after store depletion in control HeLa cells, which was dramatically decreased in DKO HeLa cells. Differently from our expectations, DKO Jurkat T cells showed only a modest reduction (~ 25%) in the PM occupancy of the junctions, especially after store depletion, when compared to control cells. While the DKO Jurkat T cells showed a proportional decrease in the number of junctional contacts, the individual junctional tubule length remained unaffected, when compared to control cells under both resting or store-depleted conditions (Fig. 3B). Interestingly, the decreased SOCE in DKO Jurkat T cells was not rescued by an enforced expansion of the ER-PM junctions by overexpression of a membrane cross-linker, MAPPER (membrane-attached peripheral ER) that creates artificial junctions with 10–25 nm distance between the ER and the plasma membranes (Fig. 3C)^16^. Contrary to the expression of MAPPER, expression of STIM1 almost fully rescued SOCE in DKO Jurkat T cells. Together, these results suggest that while E-Syts are important membrane-tethering factors in both HeLa and Jurkat T cells, they play a selective role in SOCE in Jurkat T cells, probably via regulating STIM1 function.

**Figure 4:**
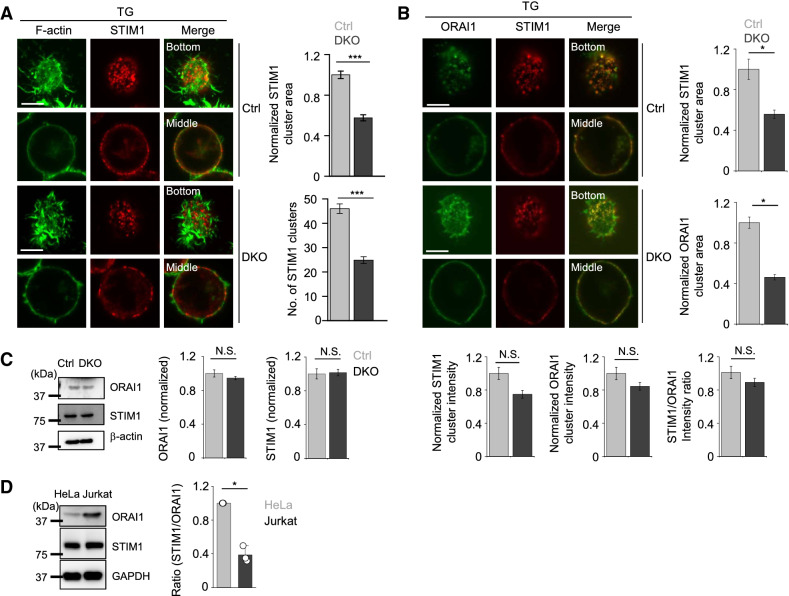
E-Syts regulate recruitment of STIM1 to the ER-PM junctions. (**A**) Representative confocal images of endogenous STIM1 in control and DKO Jurkat T cells expressing GFP-tagged F-actin under resting conditions (left) and store depletion with TG (right) (Scale bars: 5 μm). Bar graphs show normalized cluster area (top) and the number of STIM1 clusters (below) (Ctrl: n = 9, DKO: n = 8). ***p < 0.001. (**B**) Representative confocal images of endogenous STIM1 in control and DKO Jurkat T cells stably expressing low levels of ORAI1-YFP in resting and store-depleted conditions (Scale bars: 5 μm). Bar graph shows normalized STIM1 (top) and ORAI1 (middle) clustering area, STIM1 and ORAI1 cluster intensity (below—left two panels) and STIM1/ORAI1 intensity ratio (below—right panel) (Ctrl: n = 5, DKO: n = 5). *p < 0.05. *N.S.* not significant. (**C**) Expression of ORAI1 and STIM1 in control and DKO Jurkat T cells. The bar graph shows densitometry analysis of expression levels of ORAI1 and STIM1 (normalized with β-actin) from two independent experiments. *N.S.* not significant. (**D**) Expression of ORAI1 and STIM1 in HeLa S3 and Jurkat T cells. Representative immunoblots showing levels of endogenous ORAI1 and STIM1 proteins in HeLa S3 and Jurkat T cells. Glyceraldehyde 3-phosphate dehydrogenase (GAPDH) was used as a loading control. The bar graph on the right shows densitometry analysis of the ratios of STIM1 to ORAI1 (normalized to that of GAPDH) from three independent cellular lysates. *p < 0.05.

To understand the underlying molecular mechanism, we examined the clustering of endogenous STIM1 to the junctions after store depletion and found a ~ 50% reduction in number and area of STIM1 clusters in DKO Jurkat T cells (Fig. 4A). To check how reduced STIM1 translocation to the ER-PM junctions impacted SOCE, we examined co-localization between ORAI1 and STIM1 in control and DKO Jurkat T cells after store depletion. In these experiments, we used control and DKO Jurkat T cells stably expressing very low levels of ORAI1-YFP due to a lack of antibodies that reliably stain endogenous ORAI1 with a high signal/noise ratio. Consistent with reduced STIM1 translocation, recruitment of ORAI1 into the junctions in DKO Jurkat T cells was also decreased after store depletion (Fig. 4B). We did not observe any change in endogenous STIM1 or ORAI1 expression between control and E-Syts DKO cells (Fig. 4C), suggesting that E-Syts mediate SOCE via regulating recruitment of STIM1 to the ER-PM junctions in Jurkat T cells. The ratio of STIM1 vs. ORAI1 has an impact on CRAC channel gating as well as ion selectivity^23–25^. Interestingly, we found that STIM1: ORAI1 ratio in Jurkat T cells was lower than that in HeLa cells due to abundant expression of ORAI1 (Fig. 4D). Therefore, it is possible that low STIM1: ORAI1 ratio in Jurkat T cells makes them dependent on accessory factors, including E-Syts, that regulate translocation of STIM1 to the junctions.

### E-Syt2S recruits STIM1 to the ER-PM junctions via direct interaction

**Figure 5:**
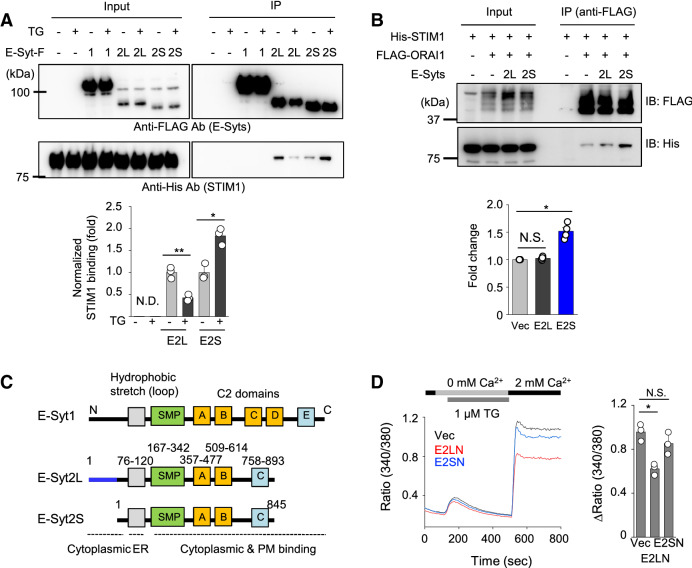
Interaction between E-Syt2S and STIM1. (**A**) Interaction of E-Syts with STIM1. FLAG-immunoprecipitates from lysates of HEK293T cells expressing FLAG-tagged E-Syt1, E-Syt2L, or E-Syt2S together with His-tagged STIM1 were blotted for detection of the indicated proteins. Cells were treated with thapsigargin before lysis (1 μM TG for 10 min) in Ringer solution containing 2 mM Ca^2+^. Bar graph shows densitometry analysis of fold changes in STIM1 band intensity (normalized to individual immunoprecipitates) from three independent experiments. *p < 0.05, **p < 0.005, *N.D* not detected. (**B**) Interaction of ORAI1 and STIM1 in the presence of E-Syt2L or E-Syt2S. FLAG-immunoprecipitates from lysates of HEK293T cells expressing FLAG-tagged ORAI1 together with His-tagged STIM1 were blotted for detection of the indicated proteins. Lysates of HEK293T cells expressing E-Syt2L or E-Syt2S were included in the binding reaction separately (see “Materials and methods" section). The bar graph shows densitometry analyses of fold changes in STIM1 band intensity (normalized to individual immunoprecipitates) from four independent experiments. *p < 0.05, *N.S.* not significant. (**C**) Schematic showing domain structure of E-Syt1, E-Syt2L and E-Syt2S. Grey boxes indicate transmembrane (TM) segments that span the ER membrane. Cytoplasmic region contains an N-terminal short fragment, a synaptotagmin-like mitochondrial lipid-binding protein (SMP) domain, and multiple C2 domains involved in targeting proteins to the plasma membrane. E-Syt2S lacks the first 48 amino acids present in E-Syt2L. (**D**) Measurement of SOCE in Jurkat T cells expressing N-terminus of E-Syt2L (E2LN) or E-Syt2S (E2SN). Intracellular stores were passively depleted with thapsigargin (1 μM, TG) in Ca^2+^-free solution, and SOCE was measured by perfusion with 2 mM Ca^2+^-containing solution. Traces show averaged SOCE responses from 30 to 50 cells, and bar graph shows averaged SOCE response ± S.E.M. from three independent experiments. *p < 0.05, *N.S.* not significant.

STIM1 is recruited into the ER-PM junctions via both intrinsic and extrinsic mechanisms; by binding of its C-terminal poly(K) residues to PIP_2_ at the PM, and via interaction with ORAI1 and other junctional proteins, including junctate and junctophilin-4^6,7,26–29^. PIP_2_ needs to be replenished upon its depletion by phospholipase C (PLC)-mediated hydrolysis, in which E-Syts play a major role. Consistently, several reports have validated a role of E-Syt1 in STIM1 recruitment into the PIP_2_-enriched ER-PM junctions in various cell types^15–18^. Hence, it is likely that E-Syts regulate PIP_2_ enrichment at the junctions in T cells as well. However, since we identified E-Syts as interacting partners of STIM1 from affinity protein purification, we examined possible interaction between E-Syts and STIM1 by co-immunoprecipitation (co-IP). Under resting conditions there was detectable binding of STIM1 with both E-Syt2L and E-Syt2S, while E-Syt1 did not show any interaction (Fig. 5A). After store depletion, STIM1 binding to E-Syt2S was enhanced, whereas that with E-Syt2L was reduced. Under similar conditions, E-Syts did not show any interaction with ORAI1 (Fig. S3A). Interestingly, although E-Syt2S did not directly interact with ORAI1, it influenced STIM1-ORAI1 interaction. Addition of E-Syt2S, but not E-Syt2L to cellular lysates containing ORAI1 and STIM1, significantly increased STIM1-ORAI1 association (Fig. 5B). These results suggest that E-Syt2S, not E-Syt2L, recruits STIM1 to the junctions and enhances its interaction with ORAI1.

E-Syts contain an ER membrane integration segment, a cytosolic SMP domain followed by multiple PIP_2_ and Ca^2+^-binding C2 domains (Fig. 5C). SMP domains have specialized function in lipid exchange^12,13^. E-Syt2 and E-Syt3 are ER-resident proteins that constitutively interact with the PM, while E-Syt1 requires elevation of cytosolic Ca^2+^ for interaction with the PM^14,15^. E-Syt2L is larger by 48 amino acids in its N terminus than E-Syt2S, with the remainder of the protein sequence being identical to E-Syt2S. GST pulldown analysis using STIM1 fragments as bait showed that E-Syt2S interacted with the serine/threonine-rich domain of STIM1, which plays a regulatory role in STIM1 function due to its location in close proximity to the ORAI1-interacting/gating domain (amino acids 342–448)^28,29^ (Fig. S3B). We were not able to detect any interaction between E-Syt1 and STIM1 in these GST pulldown assays, in consistence with the previous co-IP results. Reversely, GST pulldown assay using E-Syt2S domains as bait showed that STIM1 interacted with the linker region between the C2B and C2C domains of E-Syt2S. GST pulldown assays using E-Syt2L N terminus (E2LN) and E-Syt2S N terminus (E2SN) showed that E2LN strongly homo-multimerizes and also interacted with multiple regions of E-Syt2, including the SMP and linker domains, while E2SN did not show any interaction (Fig. S3C). Since E2LN interacted with the linker domain that binds to STIM1, it is possible that intramolecular interaction between E2LN and the linker domain may inhibit STIM1 binding. A similar auto-inhibitory mechanism has been demonstrated by interaction between C2C and C2E in E-Syt1, that is weakened by Ca^2+^ binding to C2C domain^30^. To check functional differences between E2LN and E2SN, we checked their influence on SOCE. We observed that overexpression of E2LN, but not E2SN, significantly decreased SOCE in Jurkat cells (Fig. 5D). These results further support a potential role of E2LN in auto-inhibition.

**Figure 6:**
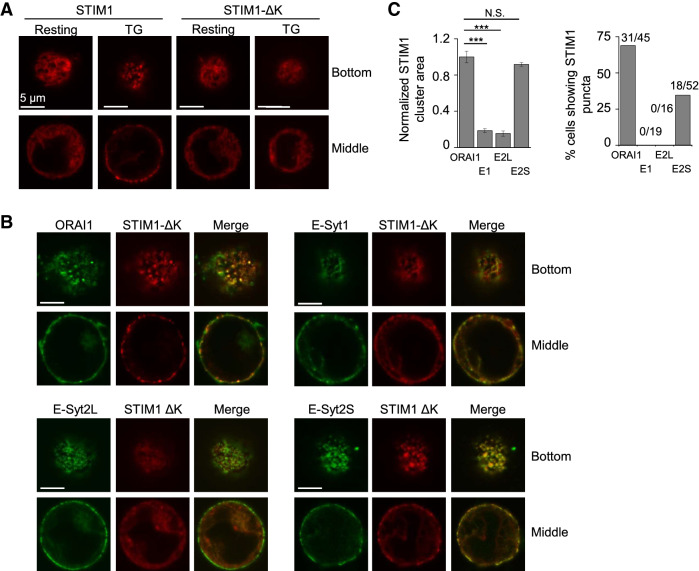
E-Syt2S mediates STIM1 recruitment to the ER-PM junctions. (**A**) Representative confocal images of Jurkat T cells expressing STIM1-mCherry and STIM1-ΔK-mCherry under resting and store-depleted conditions. (Scale bars: 5 μm). (**B**) Representative confocal images of Jurkat cells expressing STIM1-ΔK-mCherry together with ORAI1-YFP, E-Syt1-GFP, E-Syt2L-GFP, or E-Syt2S-GFP after store depletion with thapsigargin (TG) in Ringer’s solution containing 2 mM Ca^2+^ (scale bars: 5 μm). (**C**) The left bar graph shows normalized STIM1 clustering area in cells expressing STIM1-ΔK-mCherry together with ORAI1-YFP (N = 8 cells), E-Syt1-GFP (N = 8 cells), E-Syt2L-GFP (N = 6 cells), or E-Syt2S-GFP (N = 6 cells) after store depletion with thapsigargin. ***p < 0.001, *N.S.* not significant. The right Bar graph shows percentage of cells showing STIM1 accumulation in puncta in cells expressing STIM1-ΔK-mCherry together with ORAI1-YFP (N = 45 cells), E-Syt1-GFP (N = 19 cells), E-Syt2L-GFP (N = 16 cells), or E-Syt2S-GFP (N = 52 cells) after store depletion with thapsigargin. Numbers on the top of each bar represent the number of cells showing STIM1 accumulation into puncta over the total number of cells examined.

Next, we examined the interaction between E-Syt2S and STIM1 using functional assays. As previously reported^6^, STIM1-ΔK mutant that has deletion in the C-terminal poly(K) region failed to form clusters at the ER-PM junctions in Jurkat T cells due to a loss of PIP_2_ binding (Fig. 6A). Co-expression of ORAI1 rescued the translocation of STIM1-ΔK to the junctions after store depletion via direct interaction, as previously shown (Fig. 6B)^29^. Interestingly, co-expression of E-Syt2S, but not E-Syt1 or E-Syt2L induced clustering of STIM1-ΔK at the ER-PM junctions after store depletion (Fig. 6B,C). These results show that the interaction between E-Syt2S and STIM1, that we have identified from co-IP and GST pulldown assays, has direct impact on the function of STIM1.

## Discussion

E-Syts regulate STIM1 accumulation at the ER-PM junctions due to their role in PIP_2_ replenishment^15–17^. However, so far, E-Syts have not been directly implicated in regulation of SOCE. In the current work, we found that E-Syts, especially E-Syt2S, the short isoform of E-Syt2, play a direct role in the recruitment of STIM1 to the junctions via protein interaction, and hence regulate SOCE in T cells. Our results suggest that E-Syt2S has a higher affinity for STIM1 than E-Syt2L after store depletion, due to the absence of an auto-inhibitory domain in the N terminus. The contribution of E-Syt1 can be attributed to its well-established function in the distribution and replenishment of PIP_2_ at the ER-PM junctions^16–18^ due to the absence of direct interaction with STIM1. Interestingly, it was shown that the recruitment of E-Syt1 to the junctions due to cytosolic Ca^2+^ elevation by thapsigargin treatment occurred with faster kinetics, while that of STIM1 was slower and persistent^15^. Therefore, although E-Syt1 and E-Syt2S play common and compensatory roles in the function of STIM1, they may act in different kinetics and mechanisms. For example, E-Syt1 transiently responds to cytosolic Ca^2+^ elevation and enriches PIP_2_ at the ER-PM junctions, whereas E-Syt2S recruits STIM1 and stabilizes its localization at those junctions via direct interaction.

The importance of the ER-PM junctions has been highly emphasized mostly in excitable cells due to their crucial role in regulation of Ca^2+^ dynamics^31,32^. Dyad or triad junctions are the primary sites for Ca^2+^ dynamics in cardiac and skeletal muscle cells, respectively. Specialized proteins reside within these junctions, including junctophilins, junctin, junctate, mitsugumins, and sarcalumenin^31–33^. The expression and importance of these ER-PM junctional proteins in T cells remain understudied. Our previous studies showed that homologs and isoforms of junctional proteins that are present in muscle cells are expressed in T cells^6,7^. An EF-hand-containing molecule, junctate, and junctophilin form a complex at the ER-PM junctions in T cells and play a role in recruitment of STIM1 via direct interaction. An ER-resident protein SARAF was identified as an interacting partner of STIM1 and a negative regulator of SOCE, which facilitates Ca^2+^-dependent slow inactivation of CRAC channels after being recruited into the junctions^10^. TMEM110, a multi-pass ER-resident protein, interacts with STIM1 and induces its active conformation to interact with ORAI1^8,9^. Among these molecules, the depletion of only TMEM110 decreased the density of ER-PM junctions. The current study suggests that E-Syts are also the structural components of the ER-PM junctions as well as positive regulators of SOCE in T cells, together with TMEM110. The functional redundancy between E-Syts and TMEM110 in ER-PM tethering can be a topic of future investigation; however, they show apparent differences in their localization. While TMEM110 is uniformly distributed throughout the ER membrane, two members of the E-Syt family, E-Syt2 and E-Syt3, constitutively localize to the ER-PM junctions. These observations suggest that E-Syts can act as independent membrane-tethering factors, while localization of TMEM110 to the ER-PM junctions requires support from additional proteins, including STIM1 or potentially E-Syts.

Interestingly, although deletion of *ESYT1* and *ESYT2* significantly decreased the density of the ER-PM junctions in T cells, we found that their membrane-tethering function does not play a significant role in SOCE. This conclusion is based on the observation that the density of the junctions was reduced in both HeLa and Jurkat T cells, but SOCE was decreased only in Jurkat T cells. Also, artificial expansion of the junctions by membrane cross-linking did not rescue the reduced SOCE. Since STIM1 itself can expand the ER-PM junctions after store depletion^4,22^, it is possible that the decrease in the number of ER-PM junctions observed after *ESYT* deletion may not affect SOCE. The function of E-Syts in SOCE was more closely related to the regulation of STIM1 since STIM1 expression almost entirely rescued the decrease in SOCE and also, STIM1 recruitment was significantly decreased in *ESYT* DKO Jurkat T cells. There can be two possible explanations for the unique role of E-Syts in regulation of SOCE, specifically in human T cells. First, we found that the STIM1: ORAI1 ratio is lower in Jurkat T cells than HeLa cells, due to increased expression of ORAI1. Therefore, it is likely that SOCE in Jurkat T cells is sensitive to the reduced function of STIM1, in the absence of E-Syts. Second, T cells predominantly express E-Syt2S, while E-Syt2L is the major isoform in HeLa cells. Protein interaction studies showed that E-Syt2S-STIM1 interaction was enhanced when ER Ca^2+^ stores are depleted. These data suggest that E-Syt2S may be involved in recruitment of STIM1 to the junctions, similar to junctate and junctophilin-4, independently from PIP_2_ binding of STIM1^6,7^. Our data suggest that direct regulation of STIM1 is the predominant role of E-Syts in T cells, compared to other known functions of E-Syts including glycolipid exchange, because proximal TCR signaling (e.g., phosphorylation of ZAP70) and downstream signaling pathways were not influenced in *ESYT* DKO cells.

Analysis of phenotypes of Jurkat T cells and human primary T cells demonstrated the physiological importance of E-Syts in regulation of the Ca^2+^-NFAT pathway and production of inflammatory cytokines. The major difference between T cells and HeLa cells was in the expression of E-Syt2S. While E-Syt2S was the predominant isoform in human T cells, HeLa cells predominantly express E-Syt2L. Mechanistically, both E-Syt2S and E-Syt2L were able to interact with STIM1, but E-Syt2L, with extra 48 amino acids in its N terminus, engages in additional intramolecular interactions. We surmise that increased intramolecular interaction due to this longer N terminus may restrict the function of E-Syt2L in regulation of SOCE. In support of this hypothesis, we observed increased interaction between ORAI1 and STIM1 upon store depletion in the presence of E-Syt2S but not E-Syt2L. Therefore, it is possible that E-Syt2S interacts with the regulatory region of STIM1 to facilitate its recruitment to the junctions and conformational change for ORAI1 interaction, whereas the E-Syt2L N terminus inhibits this function.

In summary, our current work describes the distinct role of E-Syts in establishment of the ER-PM junctions and SOCE in T cells. The E-Syt family is evolutionarily conserved through species from yeasts to humans, and its role in membrane tethering and lipid transfer has been emphasized so far. This study is significant because it reveals a cell type-specific role of E-Syts in Ca^2+^ signaling. The current study also uncovers the underlying molecular mechanism by demonstrating a direct interaction of E-Syt2S with STIM1. We showed that T cells have a unique strategy to modulate Ca^2+^ signaling, one of the fundamental signaling pathways for their activation. Our findings suggest that although the E-Syt family has a highly conserved function in establishment of the structural platform for the ER-PM junctions, it has also developed a strategy to endow more specific features to modulate signaling pathways, depending on the cell types.

## Materials and methods

### Plasmids and cells

GST-tagged truncated STIM1 fragments have been previously described^19^. The cDNAs encoding E-Syt1 and E-Syt2S were a kind gift from Dr. Pietro De Camilli (Yale University) and E-Syt2L was cloned from cDNA of Jurkat cells. The plasmid encoding MAPPER was a kind gift from Dr. Jen Liou (UT Southwestern Medical Center). FLAG- and GST-fused full-length E-Syts and their fragments were subcloned into pMSCV-CITE-eGFP-PGK-Puro and pGEX4T-1 vectors, respectively, using primers described in Supplementary Table 1. HeLa S3 and Jurkat E6-1T cell lines were purchased from American Type Culture Collection center (ATCC, Manassas, VA).

### Affinity protein purification

HeLa cells stably expressing FLAG-tagged STIM1 were used for affinity protein purification using previously described methods^19^.

### Generation of E-Syt knockdown or knockout cells

For generation of knockdown cells^7^, HEK293T cells were transfected with plasmid(s) encoding shRNA (Supplementary Table 1) and packaging vectors (pMD2.G and psPAX2, purchased from Addgene), using the calcium phosphate transfection method. Culture supernatants were harvested at 48 and 72 h post transfection and used for infection of 2.5 × 10^6^ Jurkat T cells together with polybrene (8 µg/ml) using the spin-infection method. Cells were selected with puromycin (1 µg/ml) 48 h post infection. For knockout cells^34^, HEK293T cells were transfected with plasmid(s) encoding sgRNA (Supplementary Table 1) and packaging vectors (pMD2.G and psPAX2, Addgene) using calcium phosphate transfection method. Lentiviruses encoding Cas9 were generated using the same technique. Culture supernatants were harvested at 48 and 72 h post transfection and used for infection (50% of Cas9-encoding virus + 50% of sgRNA-encoding virus) of HeLa or Jurkat T cells together with polybrene (8 µg/ml) using the spin-infection method. Cells were selected with puromycin (1 µg/ml) and blasticidin (5 µg/ml) 48 h post infection.

### Single-cell Ca^2+^ imaging and confocal microscopy

Single-cell Ca^2+^ imaging of T Cells loaded with 1 μM Fura 2-AM was performed as previously described^35^. For each experiment, 30–50 individual T cells were analyzed using OriginPro8.5 (Originlab) software. Acquisition and data analysis were performed using Slidebook (Intelligent Imaging Innovations, Inc.) and OriginPro8.5 (Originlab) software. For depletion of stores, cells were treated with 1 µM thapsigargin or ionomycin in Ca^2+^-free Ringer’s solution (unless indicated) for 5 min. TCR stimulation of Jurkat T cells for Ca^2+^ imaging was done using 10 µg/ml of soluble α-CD3 antibodies (OKT3 clone, NCI preclinical repository. Confocal laser scanning microscopy was performed using Fluoview FV10i Confocal Microscope (Olympus), images were captured with a 60 × oil objective. Images were processed for enhancement of brightness or contrast using Fluoview software.

### Immunoprecipitation and immunoblotting

For immunoprecipitation between E-Syts and STIM1, cDNA encoding FLAG-tagged E-Syt1, E-Syt2L or E-Syt2S were transfected separately with 6 × His-tagged STIM1 into HEK293T cells. For immunoprecipitation between ORAI1 and STIM1 in the presence of E-Syt2L or E-Syt2S, cDNA encoding full-length FLAG-tagged ORAI1 and 6 × His-tagged STIM1 was transfected into HEK293T cells^7,19^. Separately HEK293T cells were also transfected with GFP-tagged E-Syt2L or E-Syt2S. Transfected cells (2 × 10^7^) were lysed in lysis buffer (20 mM Tris–Cl, 2 mM EDTA, 135 mM NaCl, 10% (vol/vol) glycerol, 0.5% Igepal CA-630, Complete Protease Inhibitor Cocktail [Sigma-Aldrich], pH 7.5) and centrifuged at 100,000×*g* for 1 h before preclearing the supernatant with protein G-Sepharose. Pre-cleared lysates of HEK293T cells expressing E-Syt2L or E-Syt2S were mixed with those of HEK293T cells expressing ORAI1 and STIM1 (1:1 ratio v/v) and immunoprecipitated with anti-FLAG antibody-conjugated resin for 6 h. Immunoprecipitates were washed five times in lysis buffer and analyzed by immunoblotting. For immunoblot analyses, cells were lysed in RIPA buffer (10 mM Tris–Cl, 1% Triton X-100, 0.1% SDS, 140 mM NaCl, 1 mM EDTA, 0.1% sodium deoxycholate, and Complete Protease Inhibitor Cocktail [Sigma-Aldrich], pH 8.0) and centrifuged to remove debris. Samples were separated on 8–10% SDS-PAGE. Proteins were transferred to nitrocellulose membranes and subsequently analyzed by immunoblotting with relevant antibodies.

### Purification of recombinant proteins from *E. coli*

Full-length and fragments (a.a. 1–249, 250–400, 324–448, 400–600, and 600–685) of STIM1 and fragments of E-Syt2 (2NL, 2NS, SMP, C2A/B, linker, and C2C) were subcloned into pGEX4T-1 plasmid^7,19^. GST fusion protein expressing transformants were grown in liquid cultures and induced with isopropyl-1-thio-β-d-galactopyranoside (IPTG, 0.2 mM) at 18 °C overnight. Subsequently, cells were harvested and resuspended in lysis buffer (50 mM NaH_2_PO_4_, 500 mM NaCl, 10% glycerol, pH 8.0) containing protease inhibitors and 0.5% Triton X-100. Lysates were sonicated, centrifuged to remove debris and incubated with glutathione sepharose 4B beads for 2 h. After washing 8 times with lysis buffer, the beads were stored in lysis buffer without Triton X-100 at − 20 °C.

### GST pulldown analysis

cDNAs encoding E-Syt1-FLAG, E-Syt2L-FLAG, E-Syt2S-FLAG, 2NL-GFP (amino acids 1–80 of E-Syt2L), or 2NS-GFP (amino acids 1–30 of E-Syt2S or 49–80 of E-Syt2L) were transfected into HEK293T cells^7,19^. Transfected cells (2 × 10^7^) were lysed in lysis buffer (20 mM Tris–Cl, 2 mM EDTA, 135 mM NaCl, 10% (vol/vol) glycerol, 0.5% Igepal CA-630, protease inhibitor mixture, pH 7.5) and centrifuged at 100,000×*g* for 1 h before preclearing with protein G-Sepharose. Lysates were incubated with 20 µg of GST or GST-tagged fragments of STIM1 for 6 h in binding buffer (0.5% Igepal CA-630, 20 mM Tris–HCl, 100 mM NaCl, 2 mM EDTA, 10% glycerol, protease inhibitors, pH 7.5). Precipitates were washed five times in lysis buffer and analyzed by immunoblotting.

### Electron microscopy

After transient transfection with HRP-ER plasmid (a kind gift from Dr. Richard Lewis, Stanford), Jurkat T cells were fixed with 2% glutaraldehyde (Electron Microscopy Sciences) in 0.1 M Na cacodylate buffer (Electron Microscopy Sciences). Fixed cells were amplified with a TSA-biotin system (PerkinElmer) and ABC kit (Vector Laboratories) for 30 min each before being pre-reacted with 1 mg/ml of 3, 3′-Diaminobenzidine (Sigma-Aldrich) in Tris-buffered saline for 10 min and then reacted with DAB with 0.01% H_2_O_2_ for 30 min. After post-fixation with 1% OsO_4_ and en bloc stain with 1% uranyl acetate, cells were further processed as previously described^5^ before embedding in Embed 812 (Electron Microscopy Sciences). Cells located by light microscopy were punched out, and 50–90-nm sections were cut and mounted on formvar-coated grids and viewed with an 80-kV transmission electron microscope (T12 Quick CryoEM) equipped with a slow-scan cooled CCD camera (Gatan 2kX2k). The number or length of HRP-containing tubules located within 50 nm of the plasma membrane were measured with selected EM images where the nucleus and entire cell circumference were visible (taken at × 8,000–× 25,000 magnification) to restrict our analysis to sections cut through the middle rather than the edges of cells. Total junctional tubule length/section means percentage of summation of each length of HRP-tubules located within 50 nm of the plasma membrane per length of cell circumference.

### Knockdown in human PBMCs

Mononuclear cells were prepared from buffy coats from healthy, unidentified adult donors, obtained under federal and state regulations from the UCLA CFAR Gene and Cellular Therapy Core Laboratory. Naïve CD4^+^ T cells were isolated using MagniSort Human CD4 naïve T cell enrichment kit (Thermo-Fisher). For T cell differentiation under non-polarizing conditions (ThN), cells were activated with 10 µg/ml of plate-coated anti-CD3 antibodies and soluble anti-CD28 antibodies in T cell medium (DMEM containing 20% FBS and 1% Pen-Strep) supplemented with 20 U/ml of IL-2. For Th17 cell differentiation, cells were activated with 10 µg/ml of plate-coated anti-CD3 antibodies and soluble anti-CD28 antibodies in T cell medium (described above) supplemented with 10 ng/ml of IL-1β and 10 ng/ml of IL-23. For generation of shRNA-encoding lentivirus, HEK293T cells were transfected with plasmid(s) encoding shRNA and packaging vectors (pMD2.G and psPAX2, Addgene) using the calcium phosphate transfection method. Culture supernatants were harvested at 48 and 72 h post transfection and used for infection of T cells together with polybrene (8 µg/ml) using the spin-infection method on days 1 and 2. Three hours post infection, virus-containing medium was replaced with fresh medium. On day 2, cells were detached from plate and on day 3, cells were selected with puromycin (2.5 µg/ml) for 18 h. The antibiotic was washed away and cells cultured for 2 more days in T cell medium (supplemented with IL-2 for ThN cells). On day 6, differentiated T cells were re-stimulated with 80 nM of PMA and 1 µM of ionomycin for 5 h for cytokine analysis. Brefeldin A (1 µg/ml) was added for the last 2 h of stimulation. Subsequently, cells were fixed/permeabilized and stained using the Transcription Factor Staining Buffer Set (eBioscience) for indicated cytokines. On day 6, around 1 million cells were harvested in TriZol (Thermo Fisher Scientific) for RNA preparation to check depletion efficiency by reverse transcription and quantitative PCR.

### Real-time quantitative PCR

For real-time quantitative PCR^7,34^, total RNA from HeLa, Jurkat T cells, or primary cells was extracted using the Direct-zol RNA Prep Kit (Zymo Research) as per the manufacturer’s instructions. cDNA was synthesized from total RNA using qScript cDNA SuperMix Kit (Quantabio). Quantitative real-time PCR was performed using iTaq Universal SYBR Green Supermix (Bio-Rad) on an iCycler IQ5 system (Bio-Rad) using the primers described in Supplementary Table 1. Threshold cycles (C_T_) for all of the candidate genes were normalized to the C_T_ values for GAPDH to obtain ΔC_T_ and further normalized to the values obtained for control samples to obtain ΔΔC_T_. The specificity of primers was examined by melt-curve analysis and agarose gel electrophoresis of PCR products.

### Statistical analyses

Statistical comparisons were performed using the Mann–Whitney *U* test to assess the significance between EAE-induced groups and two-tailed Student *t* test for other analyses^36^. Differences were considered significant when *p* values were < 0.05.

## Supplementary information

Supplementary Information 1.

Supplementary Information 2.
