# Supplementary material for: The short isoform of extended synaptotagmin-2 controls Ca2+ dynamics in T cells via interaction with STIM1
Source: Sci Rep. 2020 Sep 2;10:14433. doi: 10.1038/s41598-020-71489-7 (PMC7468131; doi:10.1038/s41598-020-71489-7)
Supplement: Supplementary file 1 — Supplementary Information 1. [file 41598_2020_71489_MOESM1_ESM.pdf]

SUPPLEMENTAL INFORMATION

The short isoform of extended synaptotagmin-2 controls Ca^{2+} dynamics in T cells via interaction with STIM1

Jin Seok Woo[1], Zuoming Sun[2], Sonal Srikanth[1], and Yousang Gwack[1]

[1]Department of Physiology, David Geffen School of Medicine at UCLA, Los Angeles, CA 90095, USA.

[2]Department of Molecular Imaging & Therapy, Beckman Research Institute of City of Hope, Duarte, CA 91010, USA.

Supplementary Figure

Supplementary Figure 1. Expression of E-Syt2 isoforms in HeLa and Jurkat T cells

(**A**) Lysates from HeLa, Jurkat, and HEK293T cells overexpressing E-Syt2L or E-Syt2S were subjected to immunoblot analysis for detection of E-Syt2. The molecular weight of the endogenous E-Syt2 isoforms expressed in Jurkat T cells matches those of exogenously expressed E-Syt2L and E-Syt2S proteins without any tag.

(**B**) Schematic showing domain structure of E-Syt2a and E-Syt2b (E-Syt2L). Gray boxes indicate transmembrane (TM) segments that span the ER membrane. Cytoplasmic region contains synaptotagmin-like mitochondrial lipid-binding protein (SMP) domain and three C2 domains (A, B and C) which are involved in targeting proteins to cell membranes. Bar graph shows mRNA levels of *ESYT2a* an *ESYT2b* in HeLa and Jurkat cells. E-Syt2b is synonymous with E-Syt2L. $^{*}p < 0.05$, N.D. – not detected.

(**C**) Measurement of SOCE in control and DKO Jurkat T cells after TCR cross-linking with anti-CD3 antibody and treatment with 0.5 µM ionomycin in the presence of external solution containing 2 mM Ca^{2+}. Traces show averaged SOCE responses from 30 to 50 cells, and the bar graph shows averaged response ± S.E.M. from three independent experiments. $^{*}p < 0.05$, $^{**}p < 0.005$.

Supplementary Figure 2. Deficiency of E-Syts impairs the NFAT pathway without influencing other proximal TCR signaling pathways

(**A**) Control and DKO Jurkat T cells were stimulated with soluble anti-CD3 antibody (10 µg/ml) and cross-linking antibody (20 µg/ml) for indicated times, and lysates were analyzed by immunoblotting for

detection of phosphorylated or total ZAP70, ERK, p38, and JNK proteins. β-actin was used as a loading control. Data are representative of at least three independent experiments.

(**B**) Representative confocal images of control and DKO Jurkat T cells expressing GFP-PLCδ-PH and ORAI1-mCherry (a marker for the plasma membrane). Middle panel shows representative line scans (lines depicted in Merge panels) showing overlap between PLCδ-PH and ORAI1 signals. Bar graph on the right shows averaged ratio (± S.E.M.) of PLCδ-PH signal intensity on the PM versus the entire cell. N.S. – not significant.

Supplementary Figure 3. Interaction between E-Syt2S and STIM1

(**A**) Interaction of E-Syts with ORAI1. FLAG-immunoprecipitates from lysates of HEK293T cells expressing FLAG-tagged E-Syt1, E-Syt2L, or E-Syt2S together with His-tagged ORAI1 were blotted for detection of the indicated proteins. Cells were treated with thapsigargin before lysis (1 μM TG for 10 mins).

(**B**) Identification of domains involved in binding of STIM1 with E-Syt2S. Recombinant GST-fused fragments of STIM1 were incubated with lysates of HEK293T cells expressing FLAG-tagged E-Syt1 and E-Syt2S and immunoblotted with anti-FLAG antibody (top). Purified GST-fused fragments of E-Syt2S were incubated with lysates of HEK293T cells expressing His-tagged STIM1 and immunoblotted with anti-His antibody (bottom).

(**C**) Interaction of the N-terminus of E-Syt2L or E-Syt2S with E-Syt2 fragments. GST-fused fragments of E-Syt2 were incubated with lysates of HEK293T cells expressing GFP-tagged N-terminus of E-Syt2L (E2LN) or E-Syt2S (E2SN) and immunoblotted with anti-GFP antibody.

Supplementary Table

List of primers, shRNAs, and sgRNAs used in this study

Gene name	Forward Primer	Reverse Primer	Comments
hE-Syt1_shRNA (mature antisense)	GAGACTTATGAGGTGATGGTA		In pLKO.1 vector
hE-Syt2_shRNA (mature antisense)	GCTCGCAGAGAAACAAGCTTA		In pLKO.1 vector
hE-Syt1_pLentiguide_sgRNA	CACCGCCCTAGCCATTGCGCATCAT	AACATGATGCGCAATGGCTAGGGC	sgRNA targeting human E-Syt1
hE-Syt2_pLentiguide_sgRNA	CACCGGCTGCTGCCCGTGTACGCGC	AACGCGCGTACACGGGCAGCAGCC	sgRNA targeting human E-Syt2
pMSCV-CITE-eGFP-PGK-Puro _hE-Syt1	GCGCGGCCGCATGGAGCGATCTCCAGGA	GCGCGGCCGCGGAGCTGCCCTTGTCCTT	Sub-cloned in pMSCV-CITE-eGFP-PGK-Puro using NotI site
pMSCV-CITE-eGFP-PGK-Puro _hE-Syt2L	CCCTCGAGATGACGCCACCGTCCCGG	CGGAATTCTGTCATCGCCTGAGGCCT	Sub-cloned in pMSCV-CITE-eGFP-PGK-Puro using XhoI and EcoRI sites
pMSCV-CITE-eGFP-PGK-Puro _hE-Syt2S	CGCTCGAGATGAGCGGCGCCCGGGGC	CGGAATTCTGTCATCGCCTGAGGCCT	Sub-cloned in pMSCV-CITE-eGFP-PGK-Puro using XhoI and EcoRI sites
pEGFPN1_hE-Syt2L	CCGCTCGAGATGACGCCACCGTCCCGG	CGGAATTCGTGTCATCGCCTGAGGCCT	Sub-cloned in pEGFPN1 using XhoI and EcoRI sites
pEGFPN1_hE-Syt2LN	GCCTCGAGATGACGCCACCGTCCCGG	GCGAATTCGCTCCACGCTCAGCACGCC	Sub-cloned in pEGFPN1 using XhoI and EcoRI sites
pEGFPN1_hE-Syt2SN	GCCTCGAGATGAGCGGCGCCCGGGGC	GCGAATTCGCTCCACGCTCAGCACGCC	Sub-cloned in pEGFPN1 using XhoI and EcoRI sites
pMSCV-CITE-eGFP-PGK-Puro _hE-Syt2L_M49L	GCGCGGCACTGCGGGGCGCTGAGCGGCGCCCGGGGCG	CGCCCCGGGCGCCGCTCAGCGCCCCGCAGTGCCGCGC	Site directed mutagenesis
pGEXT4-1_hE-Syt2_LN	GCGAATTCATGACGCCACCGTCCCGG	GCCTCGAGCTATCTTTCAGTGTCTGGAAA	Sub-cloned in pGEX4T-1 using EcoRI and XhoI sites
pGEXT4-1_hE-Syt2_SN	GCGAATTCATGAGCGGCGCCCGGGGC	GCCTCGAGCTATCTTTCAGTGTCTGGAAA	Sub-cloned in pGEX4T-1 using EcoRI and XhoI sites
pGEXT4-1_hE-Syt2_SMP	GCGAATTCGCAGAATGGCTAAATAAG	GCCTCGAGCTAGACAAGTGGAACGGTGAT	Sub-cloned in pGEX4T-1 using EcoRI and XhoI

			sites
pGEXT4-1_hE-Syt2_C2A/B	GCGAATTCGGTGTTCTAAGGATACAT	GCCTCGAGCTATGGACCCGAGTTACTGAG	Sub-cloned in pGEX4T-1 using EcoRI and XhoI sites
pGEXT4-1_hE-Syt2_Linker	GCGAATTCAACAGCACCATCAAGATG	GCCTCGAGCTAAGACTGTCCCAGGGTCGT	Sub-cloned in pGEX4T-1 using EcoRI and XhoI sites
pGEXT4-1_hE-Syt2_C2C	GCGAATTCCCACTGGGGCAGATCCAG	GCCTCGAGCTACGTGAGGTCATACCACTG	Sub-cloned in pGEX4T-1 using EcoRI and XhoI sites
hE-Syt2a	CGAGGCCACAGGCAAAG	ACGCGCTCCTCGTCTTC	qPCR primer
hE-Syt2b	CTGCTGCTGCCCGTGTA	ACGCGCTCCTCGTCTTC	qPCR primer
hE-Syt1	CTGGCGGTGCTGACTTCATT	CTCGAAGGCTCCGTTCTTTCT	qPCR primer
hE-Syt2	CAAACTATCTGGTGCTTCCCAA	GGAAACCGCAACTGAGCTATT	qPCR primer
hGAPDH	ATCGTGGAAGGACTCATGACCACA	AGAGGCAGGGATGATGTTCTGGA	qPCR primer

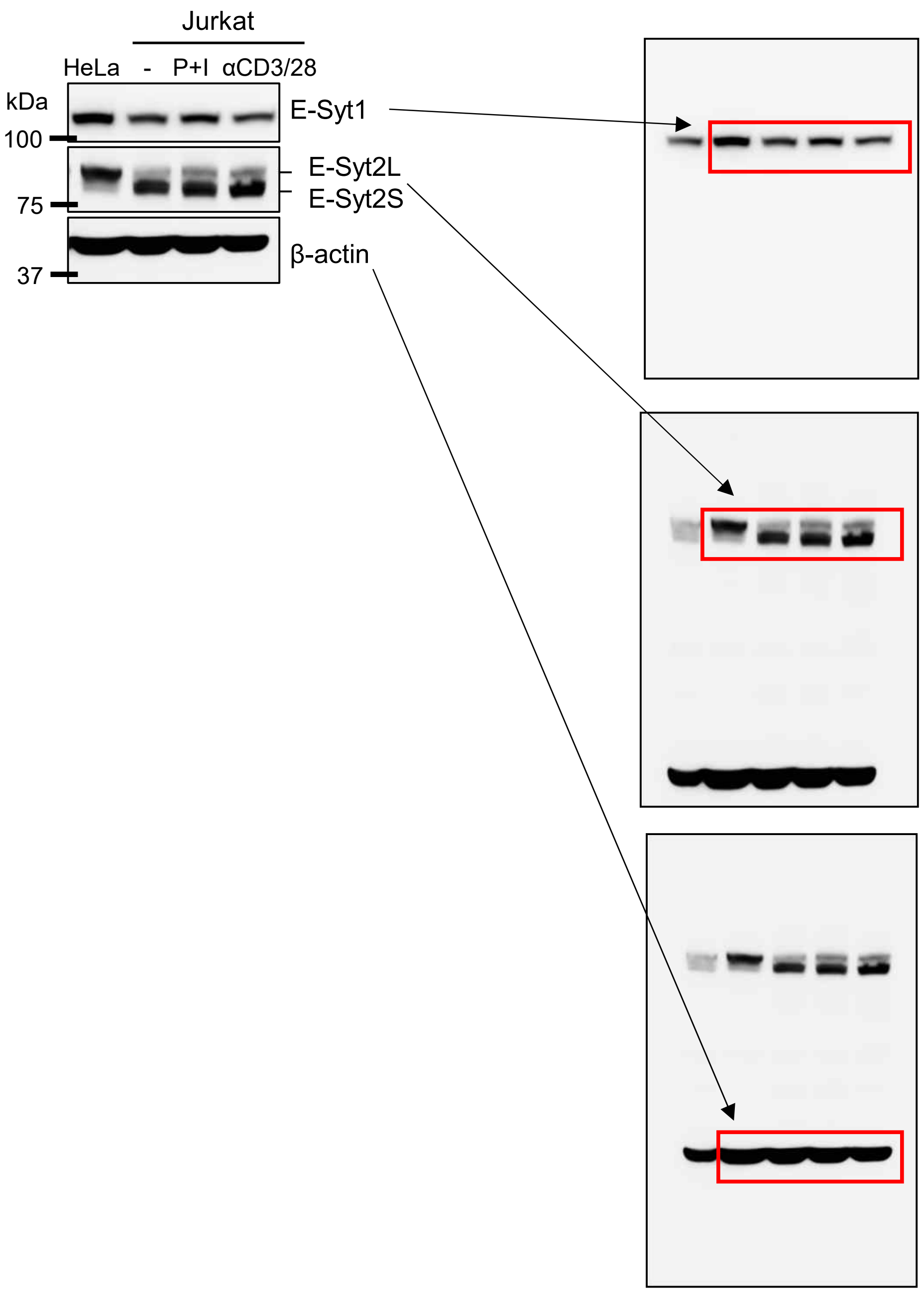

Supplementary Figure 4. Full-length blots of cropped blots from Figure 1B in the main figure.

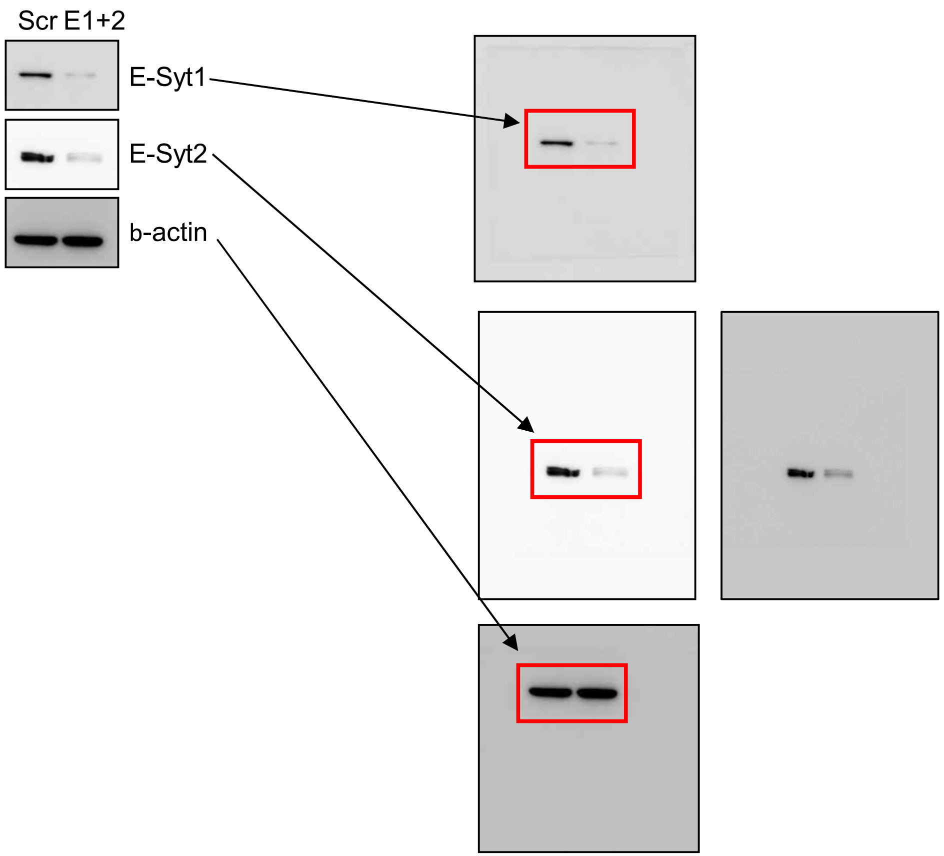

Supplementary Figure 5. Full-length blots of cropped blots from Figure 1C in the main figure.

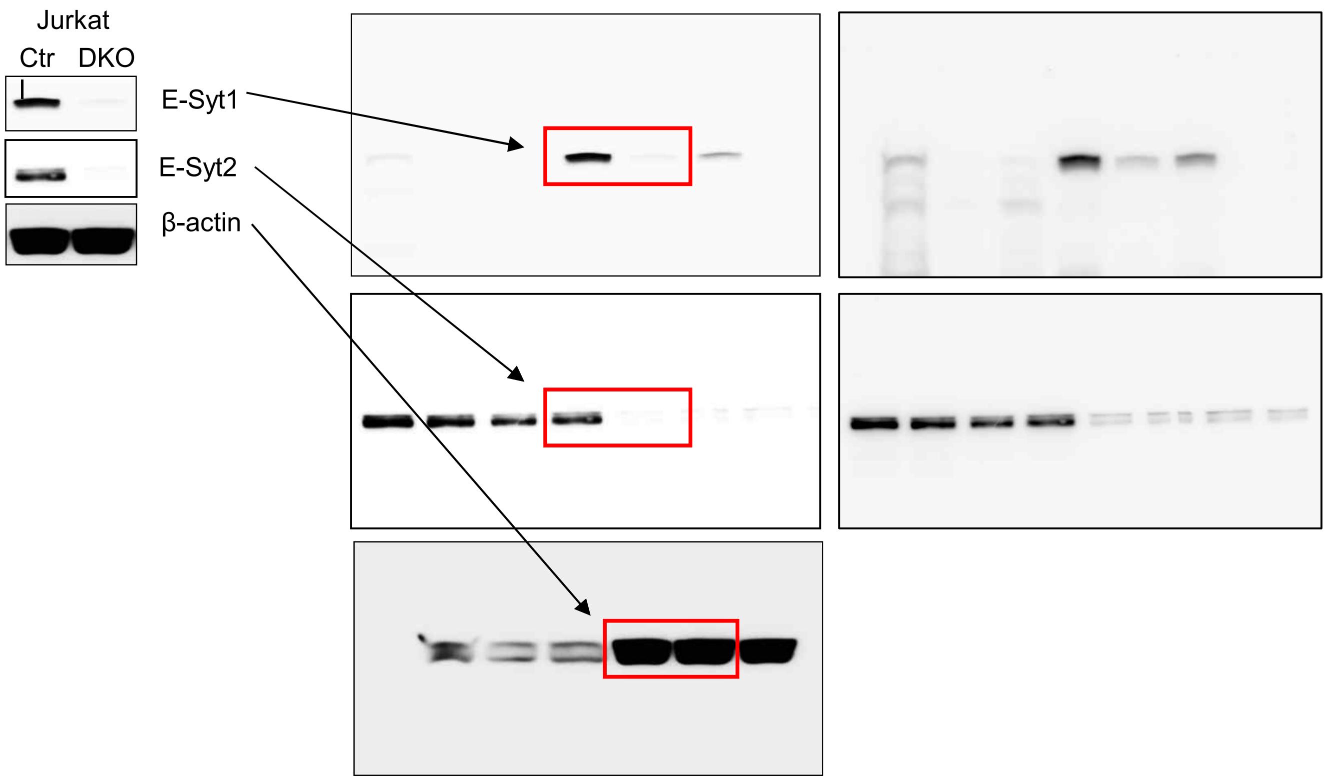

Supplementary Figure 6. Full-length blots of cropped blots from Figure 1E in the main figure.

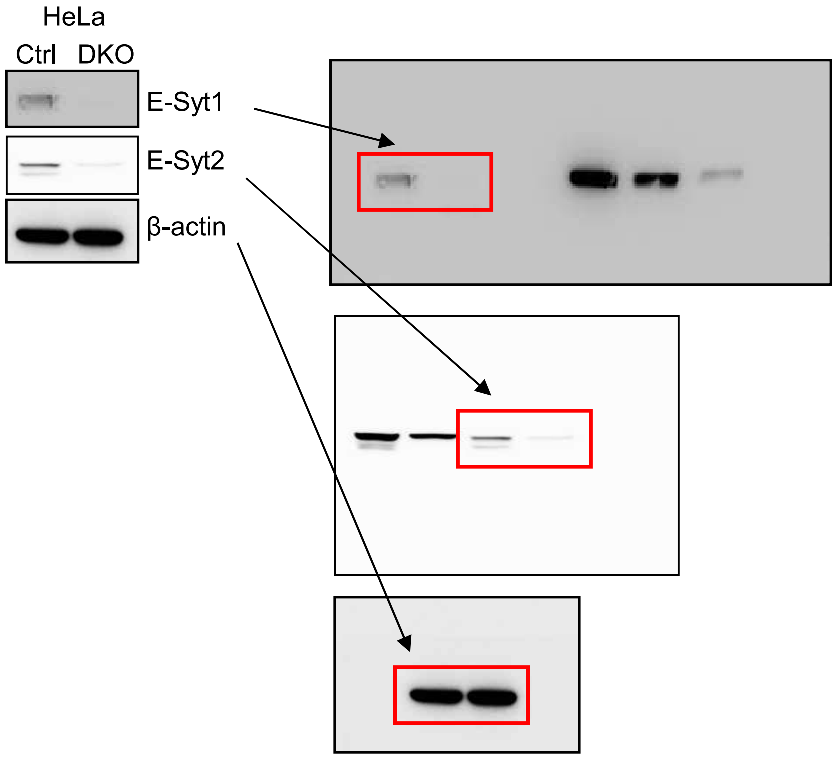

Supplementary Figure 6 (continued). Full-length blots of cropped blots from Figure 1E in the main figure.

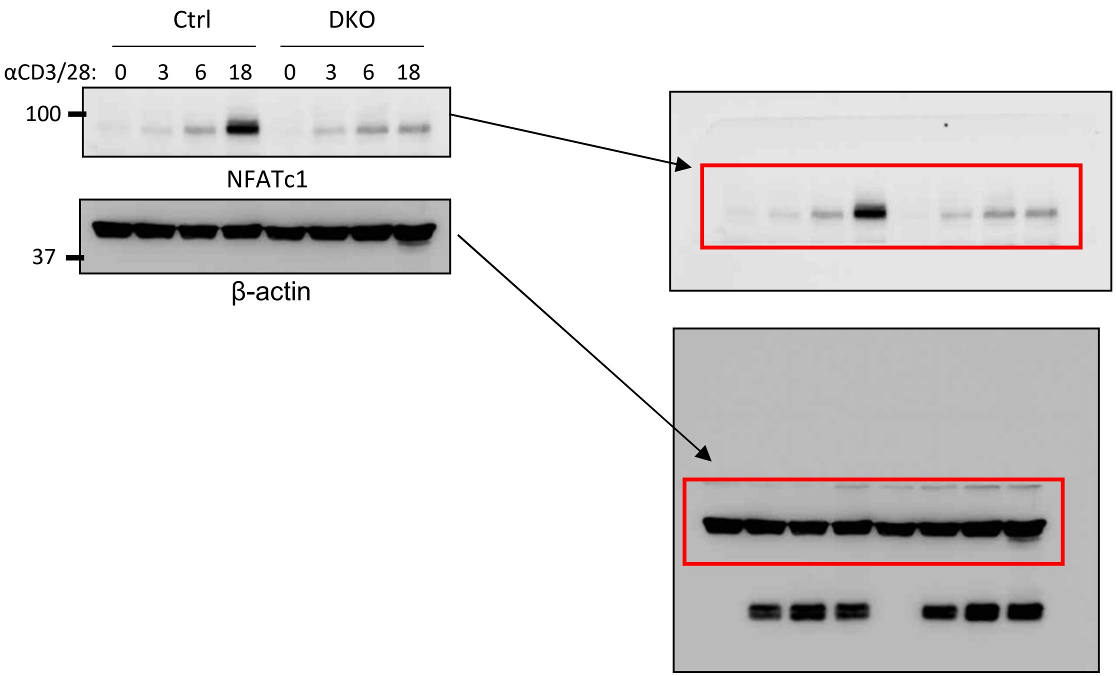

Supplementary Figure 7. Full-length blots of cropped blots from Figure 2A in the main figure.

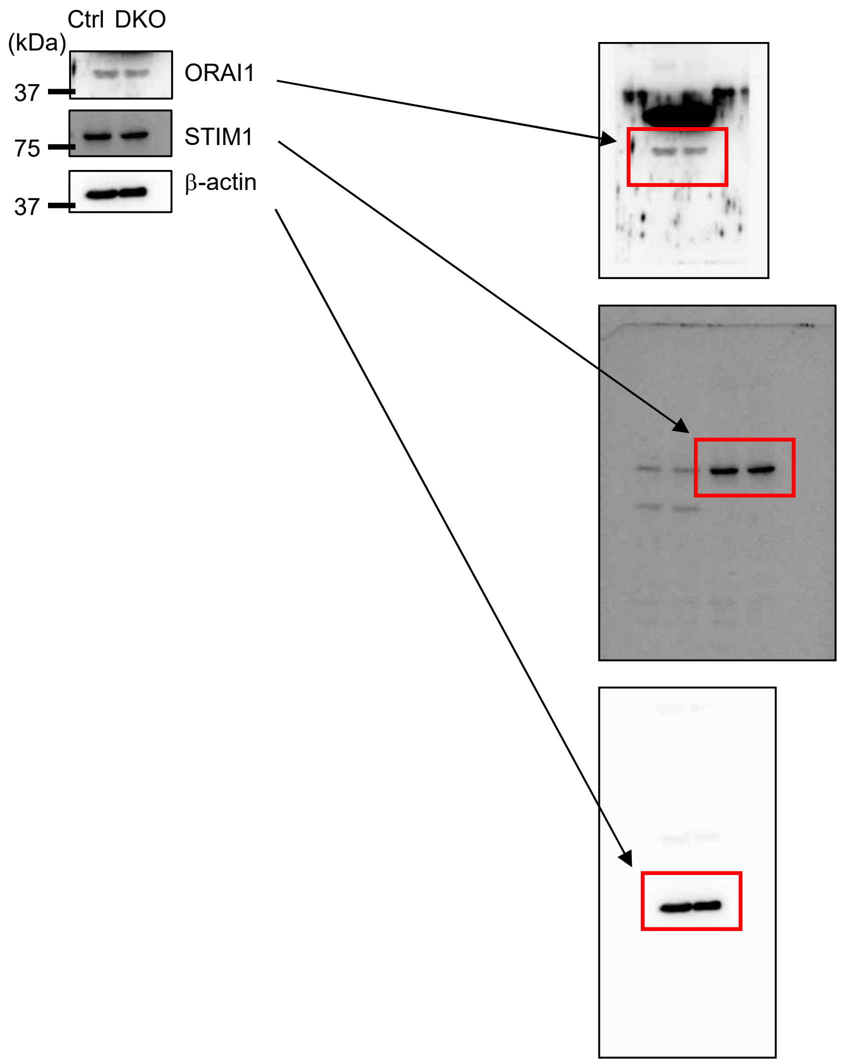

Supplementary Figure 8. Full-length blots of cropped blots from Figure 4C in the main figure.

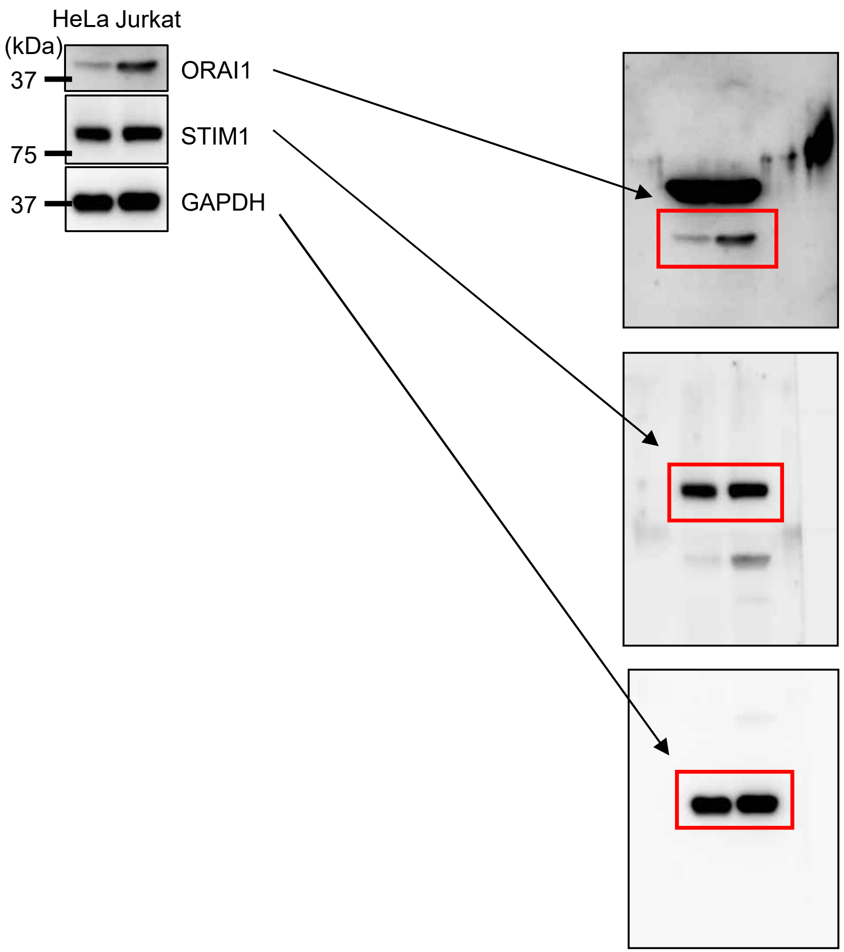

Supplementary Figure 9. Full-length blots of cropped blots from Figure 4D in the main figure.

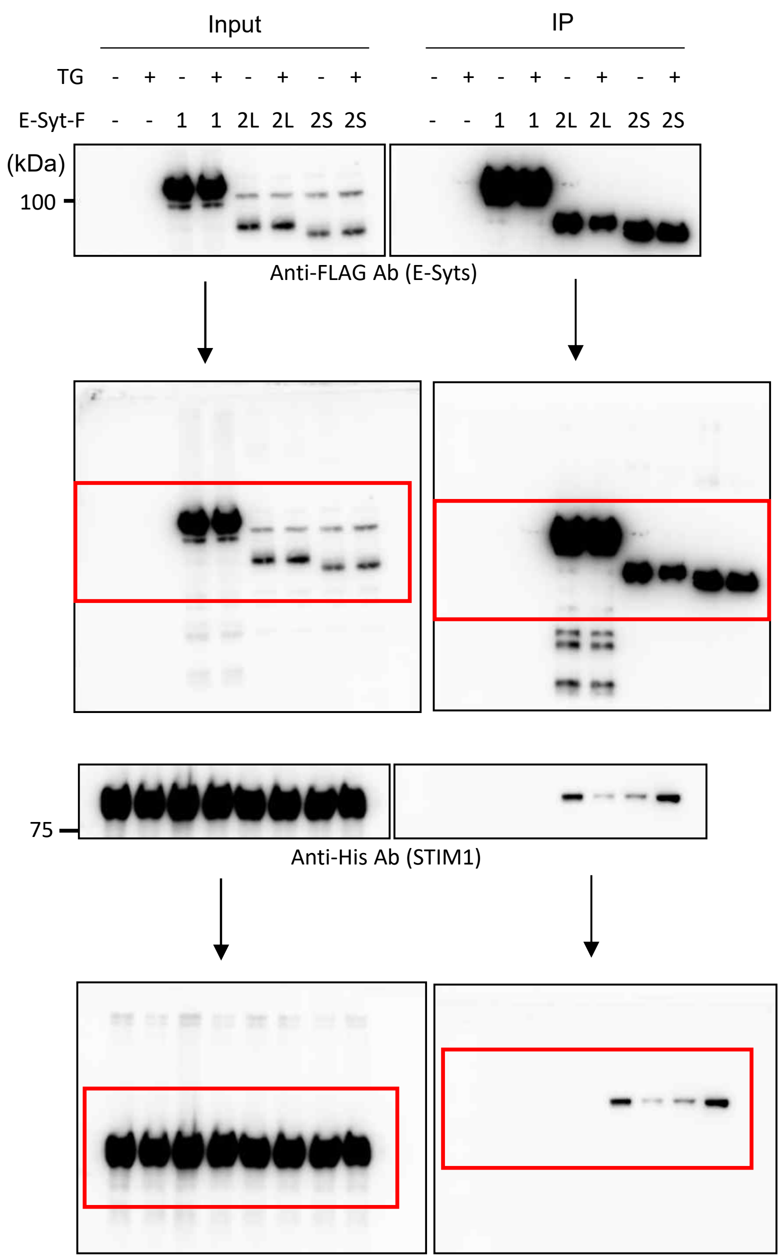

Supplementary Figure 10. Full-length blots of cropped blots from Figure 5A in the main figure.

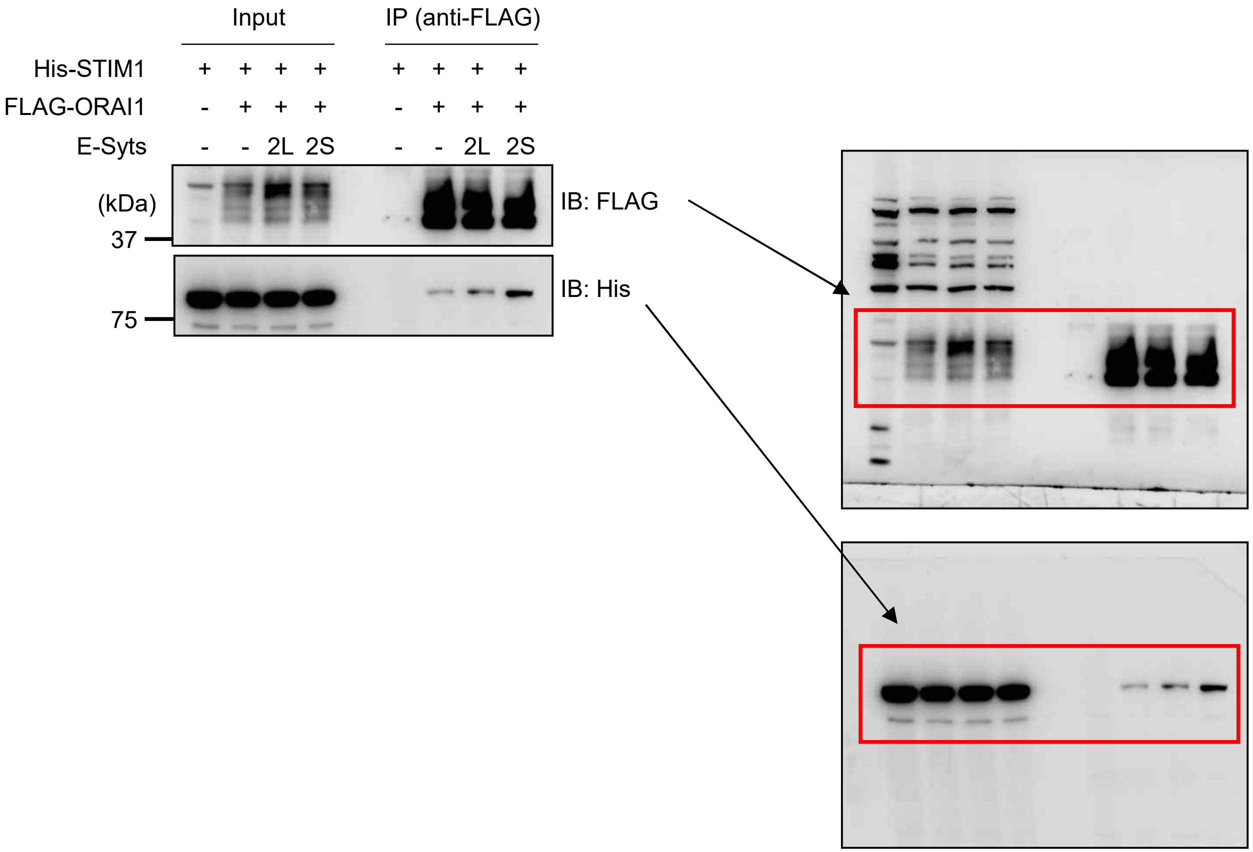

Supplementary Figure 11. Full-length blots of cropped blots from Figure 5B in the main figure.

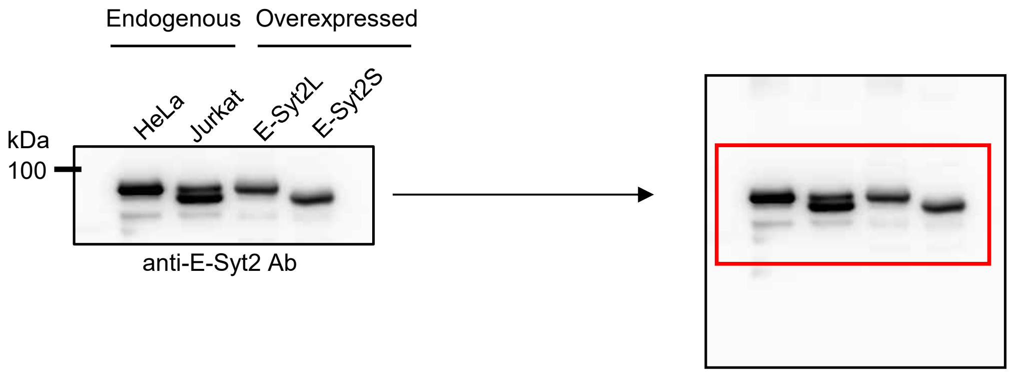

Supplementary Figure 12. Full-length blots of cropped blots from Supplementary Figure 1A.

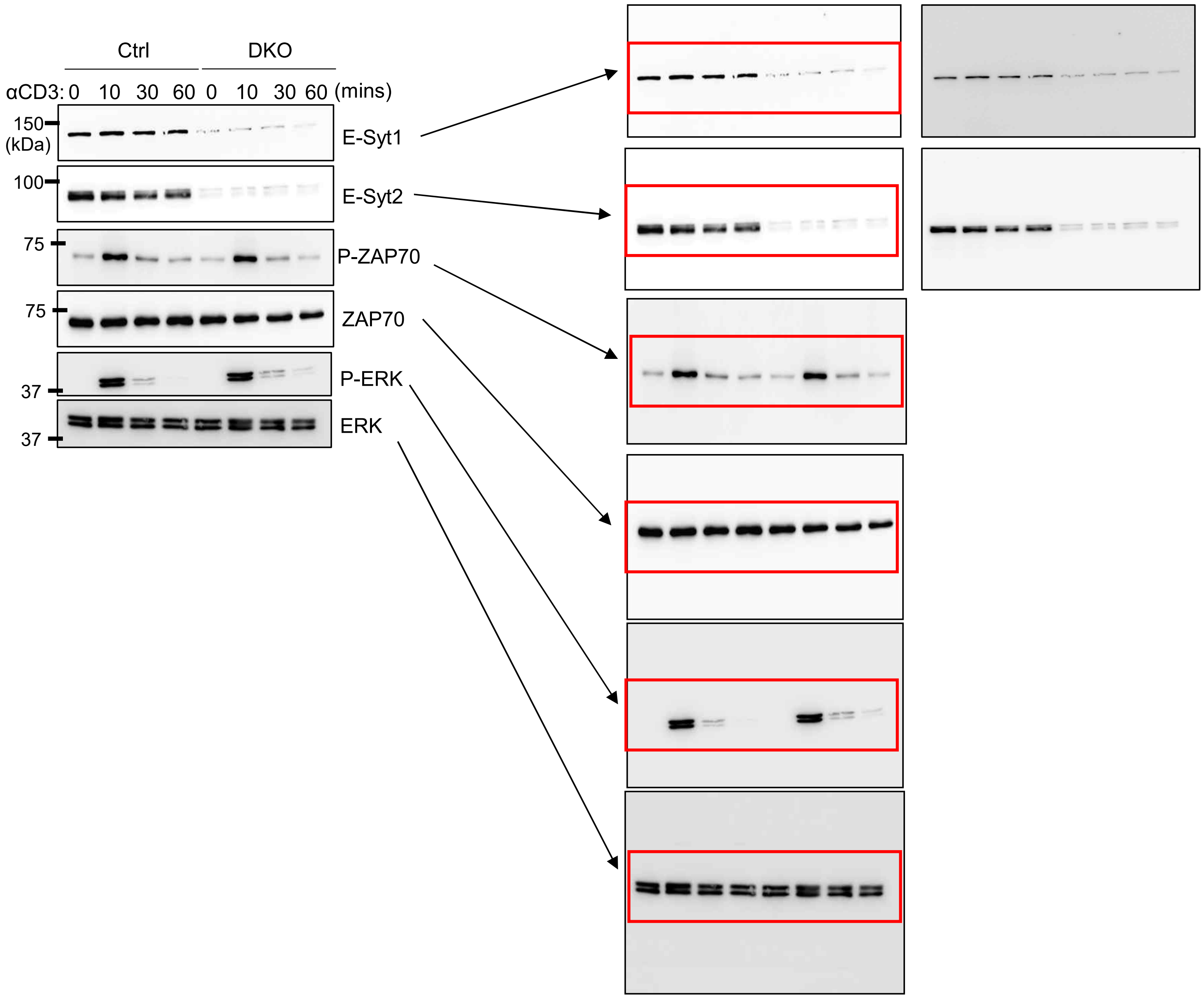

Supplementary Figure 13. Full-length blots of cropped blots from Supplementary Figure 2A.

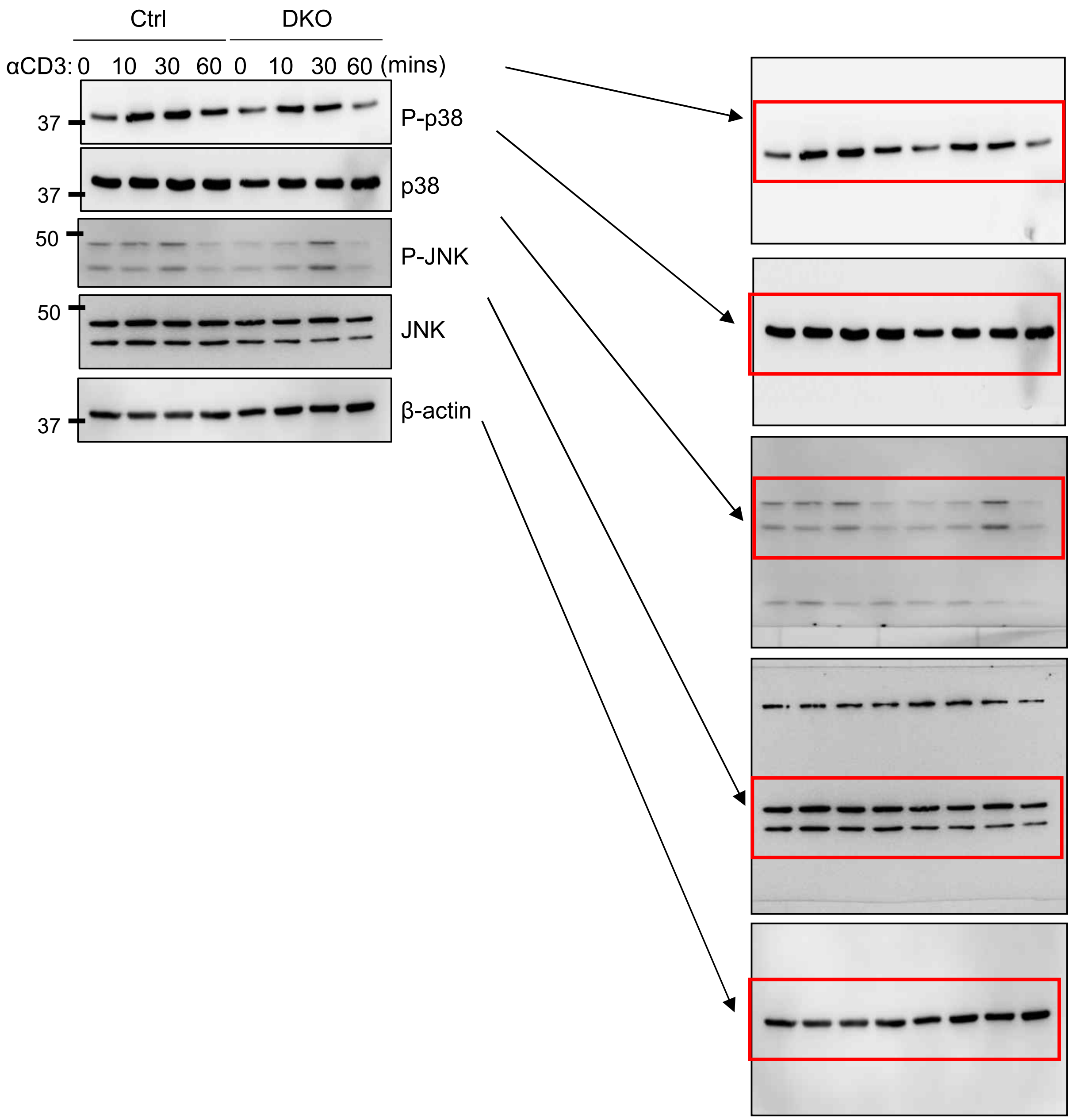

Supplementary Figure 13 (continued). Full-length blots of cropped blots from Supplementary Figure 2A.

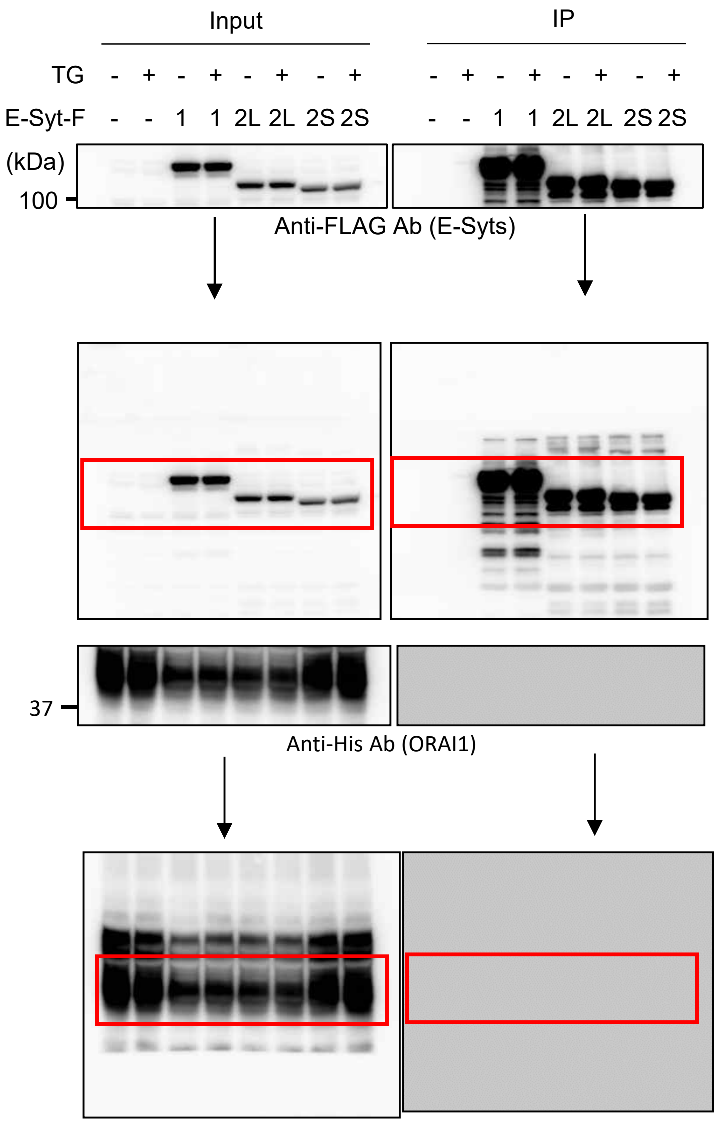

Supplementary Figure 14. Full-length blots of cropped blots from Supplementary Figure 3A.

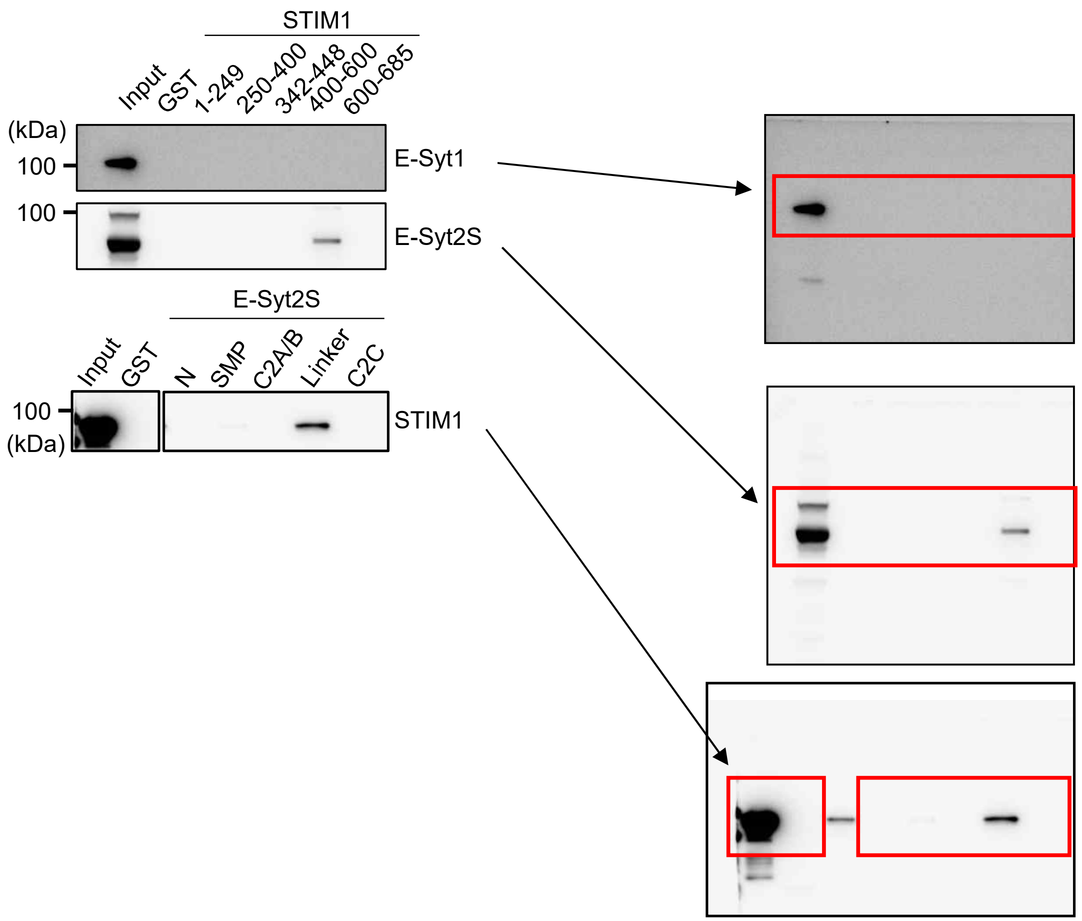

Supplementary Figure 15. Full-length blots of cropped blots from Supplementary Figure 3B.

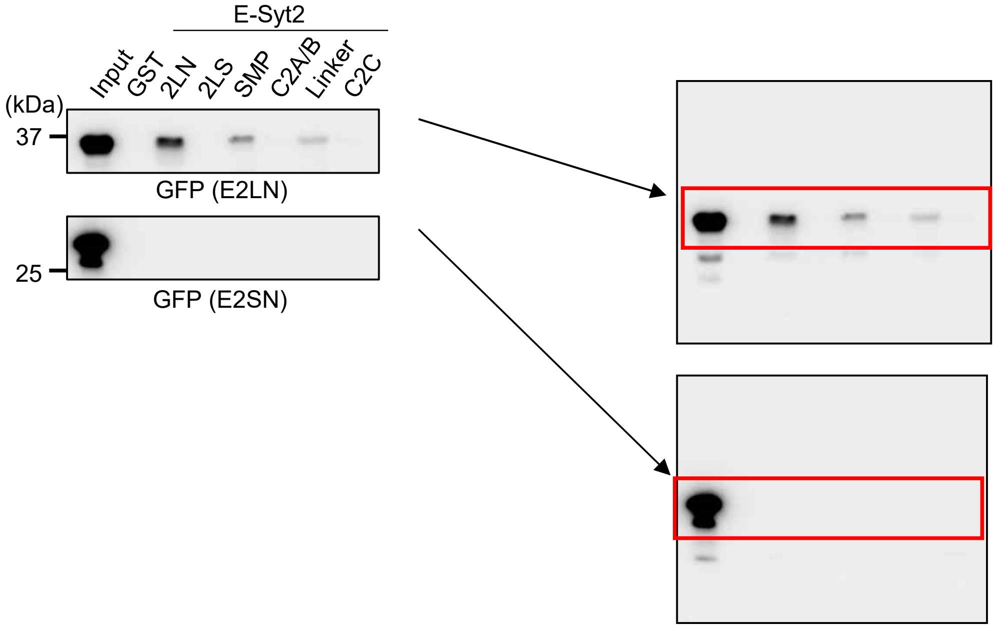

Supplementary Figure 16. Full-length blots of cropped blots from Supplementary Figure 3C.
