# Supplementary figures and images for: The short isoform of extended synaptotagmin-2 controls Ca2+ dynamics in T cells via interaction with STIM1
Source: Sci Rep. 2020 Sep 2;10:14433. doi: 10.1038/s41598-020-71489-7 (PMC7468131; doi:10.1038/s41598-020-71489-7)

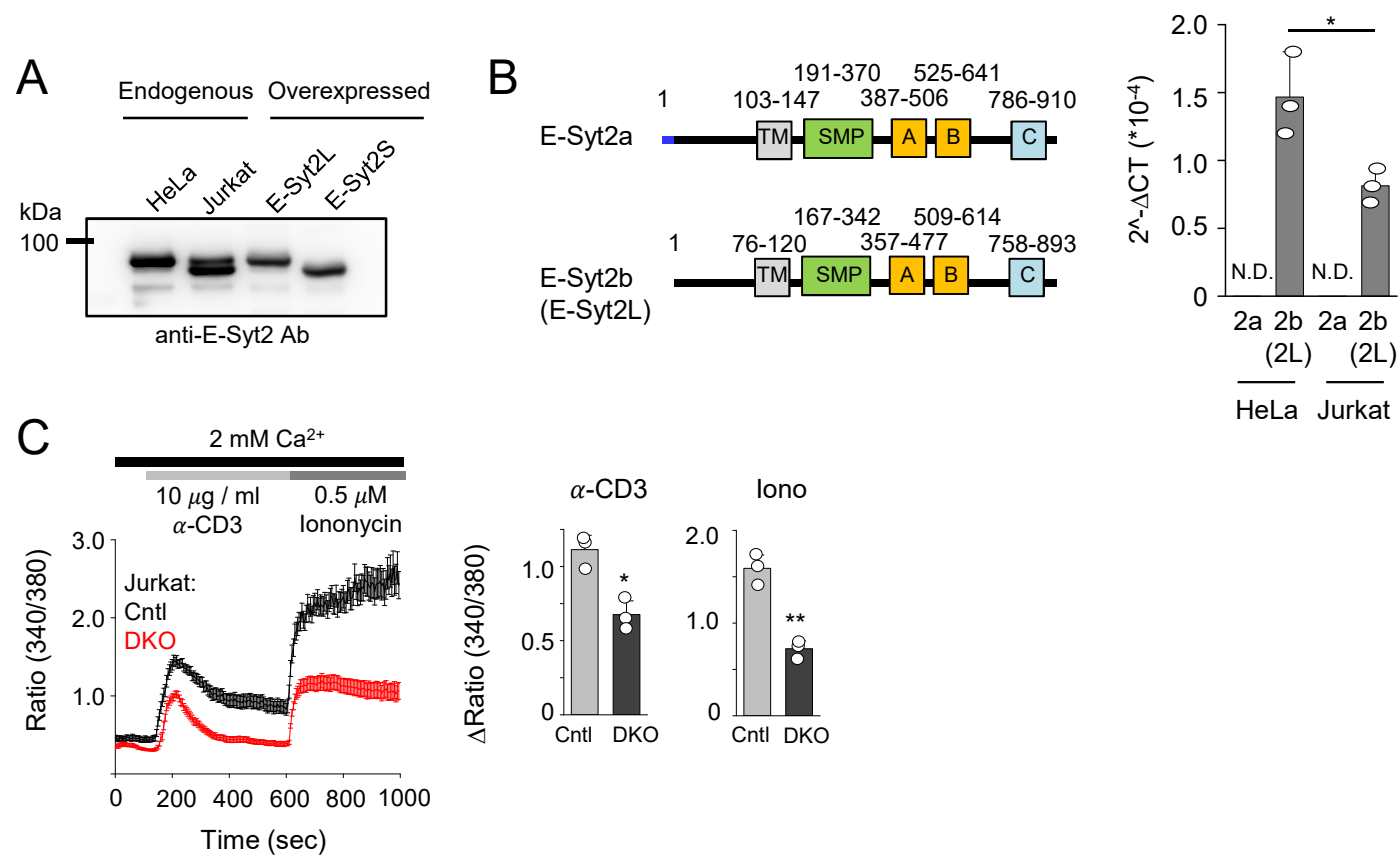

Suppl Figure 1

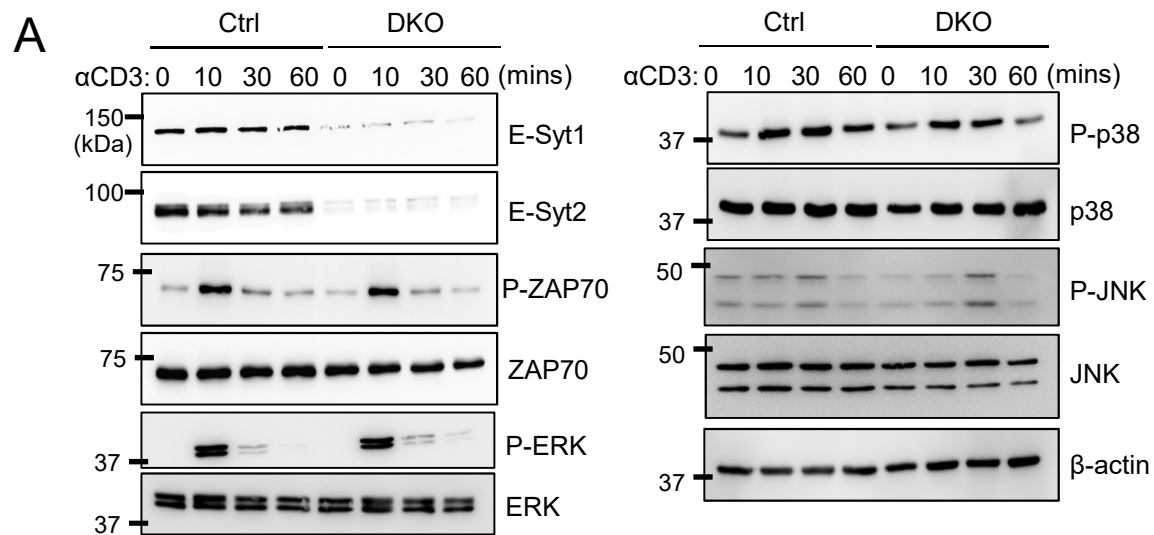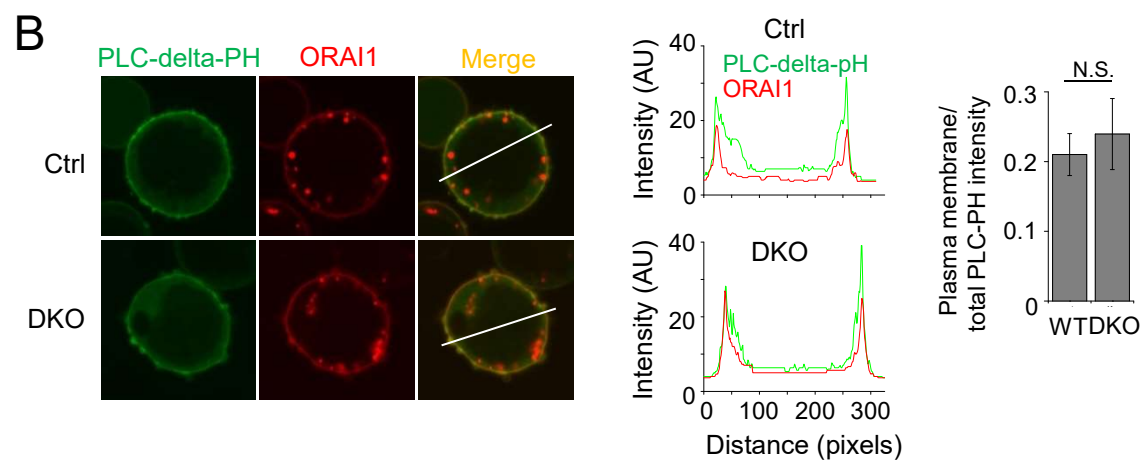

Suppl Figure 2

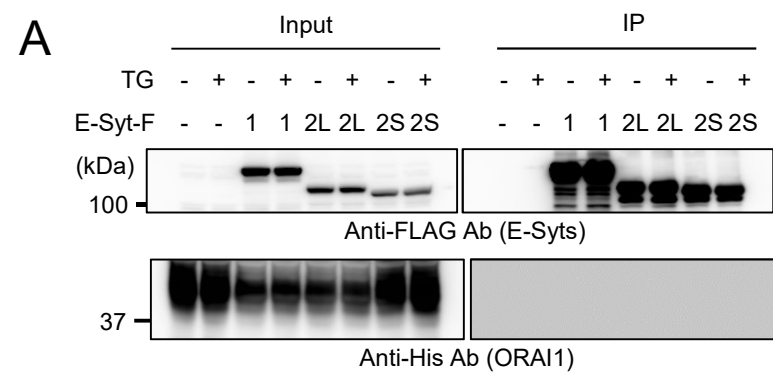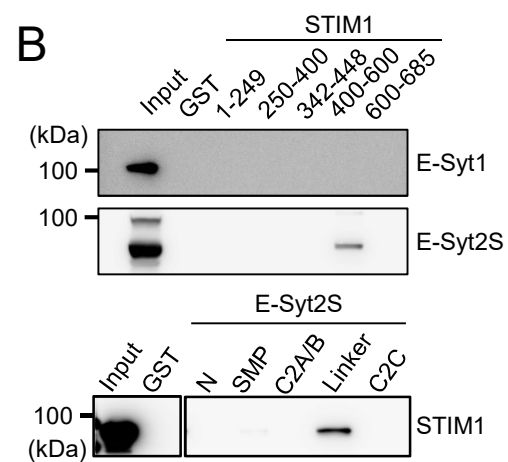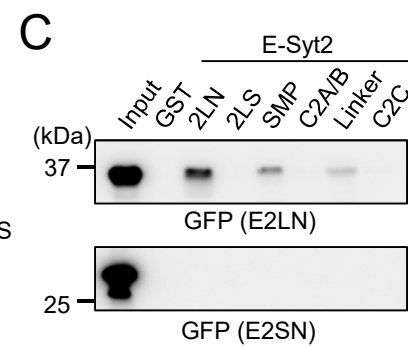

Suppl Figure 3

Supplement: Supplementary file 2 — Supplementary Information 2. [file 41598_2020_71489_MOESM2_ESM.pdf]
